# Interferon alpha-inducible protein 27 (IFI27) inhibits hepatitis B virus (HBV) transcription through downregulating cellular transcription factor C/EBPα

**DOI:** 10.1128/jvi.01509-25

**Published:** 2025-10-13

**Authors:** Xiaoyang Yu, Cheng-Der Liu, Sheng Shen, Elena S. Kim, Zhentao Liu, Hu Zhang, Ning Sun, Yuanjie Liu, Pia M. Martensen, Yufei Huang, Haitao Guo

**Affiliations:** 1 Department of Microbiology and Molecular Genetics, University of Pittsburgh School of Medicine, Pittsburgh, Pennsylvania, USA; 2 Cancer Virology Program, Hillman Cancer Center, University of Pittsburgh Medical Center, Pittsburgh, Pennsylvania, USA; 3 Department of Molecular Biology and Genetics, Aarhus University 1006, Aarhus, Denmark; 4 Department of Medicine, University of Pittsburgh School of Medicine, Pittsburgh, Pennsylvania, USA; 5 Department of Electrical and Computer Engineering, University of Pittsburgh School of Medicine 12317 , Pittsburgh, Pennsylvania, USA; St Jude Children's Research Hospital, Memphis, Tennessee, USA

**Keywords:** HBV, IFI27

## Abstract

Interferon alpha (IFNα) is the only approved immunomodulatory drug for chronic hepatitis B treatment, exerting its antiviral effects through the induction of interferon-stimulated genes (ISGs). To identify key antiviral ISGs that inhibit hepatitis B virus (HBV) replication, we performed transcriptome analysis of IFNα-treated HepG2-NTCP cells and found that IFI27 was among the most differentially expressed genes. The high inducibility of IFI27 by IFNα was further validated in primary human hepatocytes. Overexpression of IFI27 significantly suppressed HBV replication in both HBV-transfected and -infected cells, primarily by reducing HBV RNA transcription. Conversely, IFI27 knockdown markedly diminished the antiviral effect of IFNα in HBV-infected cells. IFI27 is predominantly localized in the cytoplasm, and RNA-seq analysis revealed that IFI27 inhibits HBV transcription without drastically altering the host transcriptome, indicating that IFI27 does not inhibit HBV transcription directly or through altering the transcription of cellular transcription factors or inducing antiviral signaling pathways. Instead, we found that IFI27 suppresses HBV transcription by promoting the ubiquitination-dependent proteasomal degradation of C/EBPα in the cytoplasm, a cellular transcription factor critical for HBV RNA transcription. Further investigation identified the E3 ubiquitin ligase SKP2 as a key mediator of this process, facilitating IFI27-induced C/EBPα ubiquitination and degradation. Notably, SKP2 knockdown abrogated IFI27’s antiviral activity against HBV. Taken together, our findings reveal that IFI27 contributes to IFNα-mediated antiviral activity against HBV by targeting C/EBPα for SKP2-dependent ubiquitination and proteasomal degradation. This study thus sheds new light on the antiviral mechanism of IFNα-based therapy for chronic hepatitis B.

**IMPORTANCE:**

Chronic hepatitis B virus (HBV) infection affects approximately 250 million people worldwide with limited treatment options. Interferon alpha (IFNα) remains the only approved immunomodulatory treatment for chronic hepatitis B, working in HBV-infected liver cells through inducing antiviral genes. To identify key interferon-inducible genes involved in HBV suppression, we performed transcriptome analysis of IFNα-treated liver cells and identified IFI27 as one of the most upregulated genes. Functional studies demonstrated that IFI27 inhibits HBV replication by reducing viral RNA transcription, and its knockdown significantly impaired the antiviral effect of IFNα. Mechanistically, IFI27 suppresses HBV transcription by promoting the ubiquitin-proteasome-mediated degradation of C/EBPα, a transcription factor critical for HBV RNA synthesis. This process is dependent on the E3 ubiquitin ligase SKP2, as SKP2 knockdown abolished IFI27-mediated antiviral activity. These findings reveal IFI27 as a critical mediator of IFNα-induced antiviral responses against HBV and provide new insights into host-directed antiviral mechanisms with potential therapeutic implications.

## INTRODUCTION

Despite the existence of a highly effective prophylactic vaccine, chronic hepatitis B virus (HBV) infection remains a formidable global health challenge affecting over 257 million individuals worldwide (1–3). HBV is a noncytopathic, hepatotropic virus belonging to the *Hepadnaviridae* family (4). The virion harbors a 3.2 kilobase (kb), partially double-stranded (ds), relaxed circular (rc) DNA genome (5). Upon infection by exploiting the hepatocyte-specific receptor sodium taurocholate cotransporting polypeptide (NTCP) (6), viral rcDNA is delivered into the nucleus to form an episomal covalently closed circular DNA (cccDNA) via DNA repair process (7–9). By utilizing cccDNA as the genuine template for transcription, five mRNAs with overlapping 3´ ends are transcribed, including the 3.5 kb pregenomic (pg) and precore (pC) mRNA, 2.4/2.1 kb preS/S mRNA, and 0.7 kb X mRNA (5, 10). HBV replicates its DNA genome via viral polymerase-primed and -catalyzed reverse transcription of pgRNA in the cytoplasmic nucleocapsid (11, 12). The newly synthesized rcDNA-containing nucleocapsid is either enveloped and secreted through multivesicular bodies as a virion, or it can be recycled back to the nucleus to replenish the cccDNA pool (4, 13).

Interferons (IFNs) are a group of signaling proteins produced and released by host cells in response to diverse pathogens, such as bacteria, viruses, and parasites (14). Interferon alpha (IFNα) belongs to type I IFN, which is the first remedy approved for the treatment of chronic hepatitis B and can achieve sustained virological response in a minority of patients, with significant side effects (15). Upon binding of IFNα to its cognate receptor on cell surface, it initiates a JAK/STAT signaling cascade, which leads to the induction of more than 300 IFN-stimulated genes (ISGs) (16, 17). Besides the immunomodulatory functions of some ISGs induced in immune cells, certain ISGs play antiviral activities directly in the virally infected cells (16). Various ISGs have been reported to inhibit HBV replication at different steps, primarily through both transcriptional and post-transcriptional mechanisms (18–20). For example, APOBEC3A induces cccDNA deamination and degradation (21); STAT1, SMCHD1, and PML bind to cccDNA minichromosome and shape a suppressive epigenetic status of cccDNA (22); TRIM5γ inhibits HBV transcription by promoting the degradation of HBx (23); zinc finger antiviral protein (ZAP) and ISG20 bind to HBV RNA and accelerate RNA decay (24, 25); indoleamine 2,3-dioxygenase can induce tryptophan deprivation and block HBV protein translation (26); tetherin inhibits HBV virion egress (27), etc. Identifying additional anti-HBV ISGs and elucidating their antiviral mechanisms will enhance our understanding of IFNα therapy and potentially optimize its use for treating HBV infection.

In search of ISGs that inhibit HBV replication, we have previously found that the 12 kDa ISG product, referred to as ISG12a (also known as interferon alpha-inducible protein 27 [IFI27]), inhibited HBV replication primarily through reducing HBV RNA transcript levels in cell cultures (26). This protein belongs to the FAM14 gene family, whose members encode small hydrophobic proteins that contain at least one copy of an approximately 80 amino acid conserved ISG12 motif (28). The hydrophobic nature of these proteins suggests a potential association with cellular membranes. To date, IFI27 has been reported to exert its antiviral effects against hepatitis C virus (HCV), West Nile virus, and Newcastle disease virus, utilizing different mechanisms (29–32). Furthermore, IFI27 has shown potential correlation with respiratory syncytial virus (RSV) infection due to its significant expression in clinical cases during RSV infection (33). Recently, IFI27 has also emerged as an early predictor of coronavirus disease 2019 (COVID-19). It has been reported that the expression of IFI27 was found in the respiratory tract of COVID-19 patients, and an elevated IFI27 expression in the lower respiratory tract was associated with higher viral load (34).

In this study, we aimed to further characterize the antiviral function and mechanism of IFI27 in the innate control of HBV. We report herein that (i) endogenous IFI27 acts as a restriction factor for HBV replication and is highly upregulated by IFNα to enhance its antiviral effect; (ii) IFI27 inhibits HBV replication primarily by suppressing viral promoter activity; (iii) the antiviral mechanism of IFI27 involves promoting the degradation of a cellular transcription factor (TF) C/EBPα (CCAAT-enhancer-binding protein alpha) via the ubiquitin-proteasome pathway; and (iv) the E3 ubiquitin ligase SKP2 is required for IFI27-mediated C/EBPα ubiquitination and degradation and plays a key role in IFI27-induced suppression of HBV transcription. Our findings provide new insights into IFI27 biology and its role in IFNα-based treatment of HBV infection, offering a potential opportunity for optimizing IFNα therapy and developing novel antiviral strategies against HBV.

## RESULTS

### IFI27 is highly induced in hepatocytes by IFNα treatment

**Fig 1:**
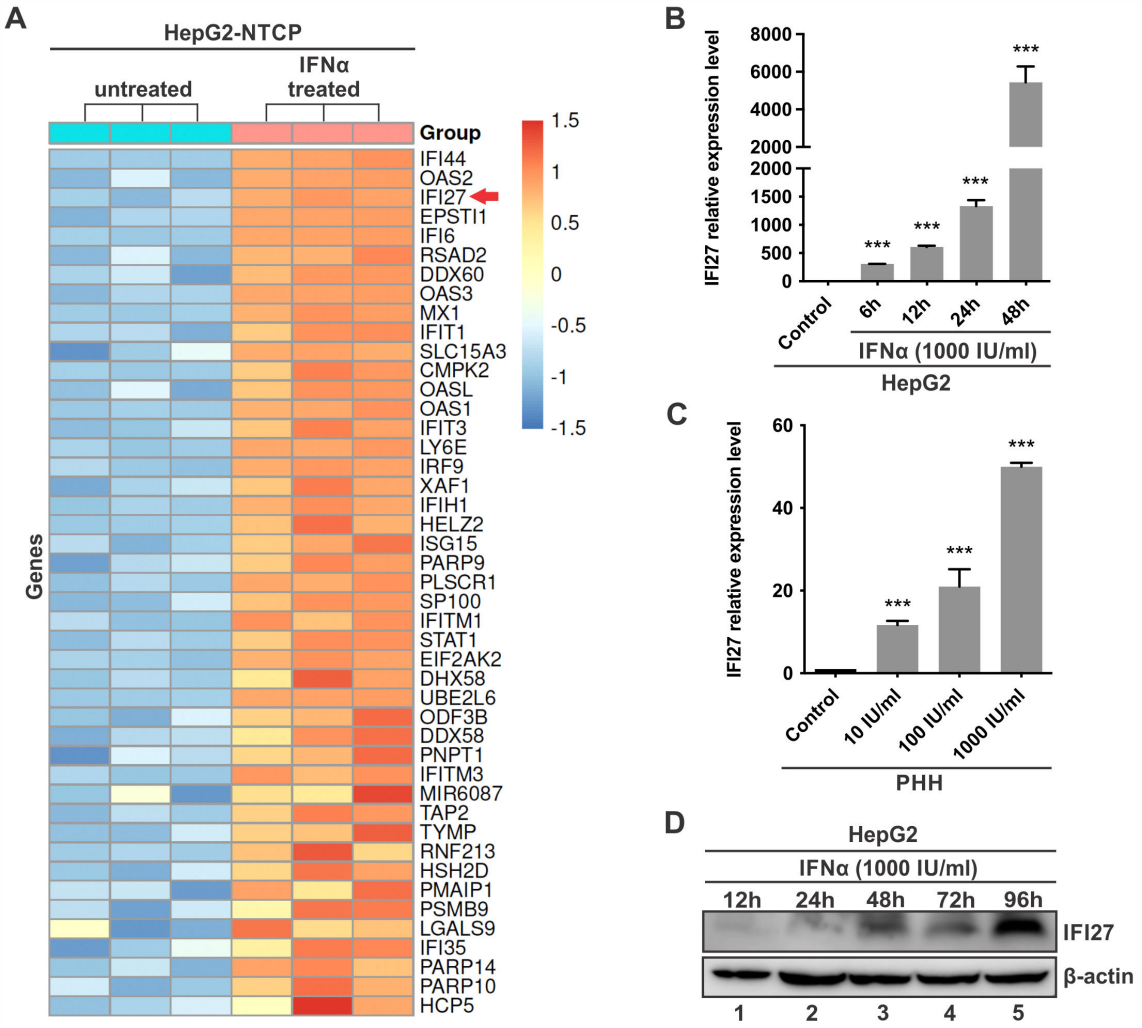
The inducibility of IFI27 by IFNα in cell cultures. (**A**) Expression heatmap of significantly upregulated ISGs in HepG2-NTCP cells by IFNα treatment. HepG2-NTCP cells were either left untreated or treated with human IFNα (1,000 IU/mL) for 36 h, followed by mRNA sequencing. Expression values for induced ISGs were Z-score normalized and are displayed as scaled values centered on 0. High and low expressions are indicated in red and blue, respectively. The IFI27 gene is indicated with an arrow. (**B**) Time course analysis of IFI27 mRNA induction by IFNα. HepG2 cells were either left untreated (control) or treated with IFNα (1,000 IU/mL) for 6, 12, 24, and 48 h. The mRNA levels of IFI27 expression were detected by RT-qPCR and normalized to β-actin. (**C**) Dose-dependent induction of IFI27 mRNA by IFNα. PHHs were either left untreated or treated with indicated concentrations of IFNα for 48 h, followed by RT-qPCR analysis of IFI27 mRNA. (**D**) Time-dependent induction of IFI27 protein expression by IFNα. HepG2 cells were treated with IFNα (1,000 IU/mL) and harvested at the indicated time points for Western blot analysis of IFI27 protein levels. β-Actin served as a loading control. Data in the histograms are presented as mean ± SD (*n* = 3), ***P < 0.001.

In our previous study to identify anti-HBV ISGs by screening a collection of over 30 ISG expression plasmids in pHBV1.3-cotransfected HepG2 cells, IFI27 emerged as a hit exhibiting potent antiviral activity (26). In the current study, to further characterize the antiviral effect of IFI27 on HBV infection and elucidate its mechanism of action, we first assessed the inducibility of IFI27 in hepatocyte-derived cells. The RNA-seq transcriptomic analysis demonstrated that IFI27 was one of the most highly induced ISGs in HepG2-NTCP cells upon IFNα treatment (Fig. 1A). Next, the inducibility of IFI27 mRNA and protein expression by IFNα was further analyzed in HepG2 and/or PHH cells, which demonstrate that IFNα induces IFI27 expression in a time- and dose-dependent manner (Fig. 1B through D). The observed less efficient IFI27 induction in PHH cells than that in HepG2 cells might be attributable to the specific batch of PHH cells used in this experiment.

### IFI27 inhibits HBV replication primarily through suppressing viral transcription

**Fig 2:**
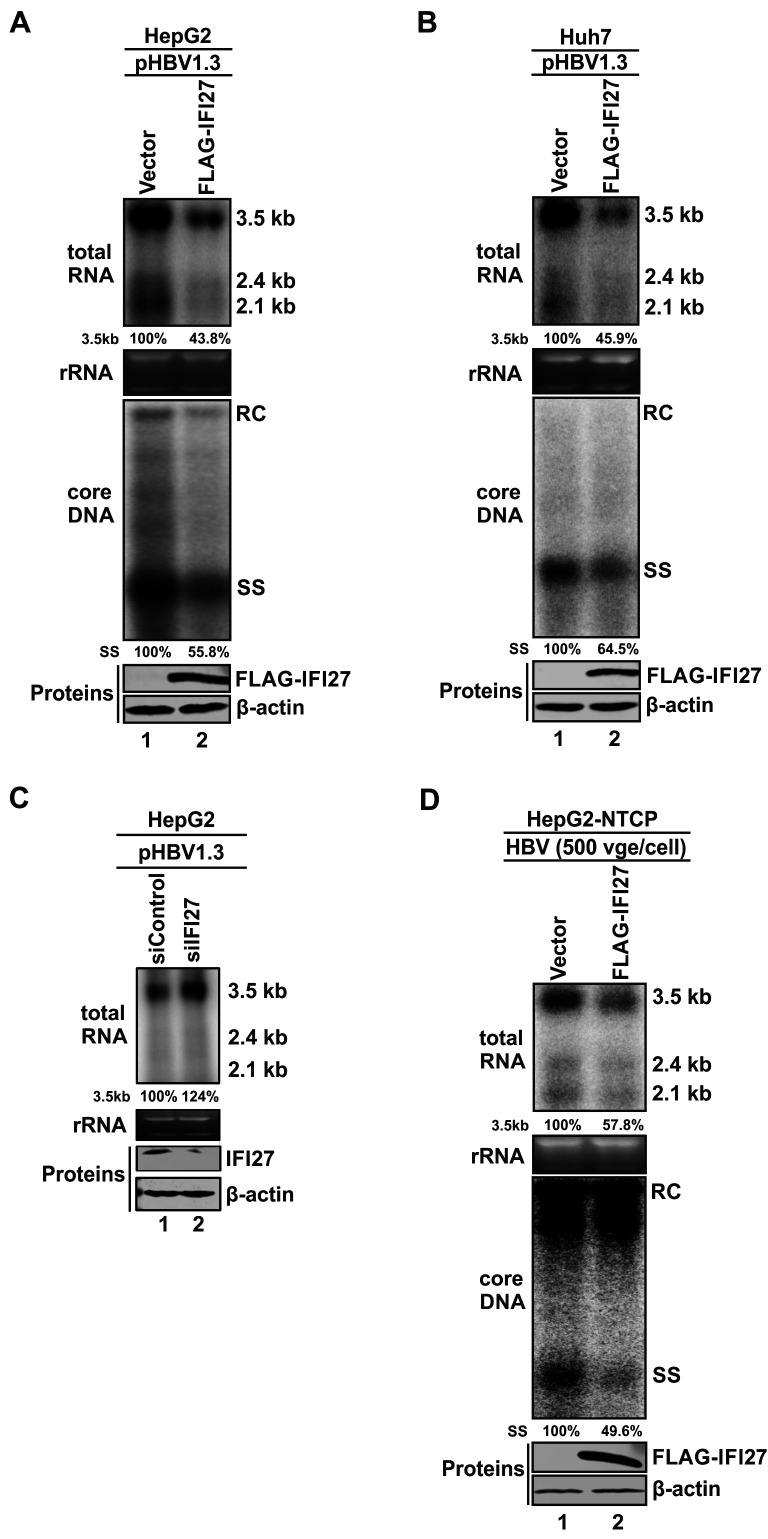
IFI27 inhibits HBV replication primarily through reducing viral RNA. (**A**) HepG2 or (**B**) Huh7 cells were co-transfected with pHBV1.3 plus either a control vector or plasmid FLAG-IFI27. Cells were harvested on day 5 post-transfection, and the levels of intracellular HBV total RNA and cytoplasmic core DNA were analyzed by Northern and Southern blot, respectively. The positions of HBV 3.5 kb pC mRNA and pgRNA as well as 2.4/2.1 kb surface mRNAs on Northern blot were labeled. Ribosomal RNA (rRNA) served as an RNA loading control. On HBV core DNA Southern blot, the positions of the relaxed circular (RC) and the single-stranded (SS) DNAs were indicated. The relative levels of HBV 3.5 kb RNA and single-stranded DNA (ssDNA) in the FLAG-IFI27 overexpression groups were shown as percentages of the control vector groups. The expression of FLAG-IFI27 was analyzed by Western blot. β-Actin served as the protein loading control. (**C**) HepG2 cells were transfected with 20 nM control siRNA (siControl) or IFI27 siRNA (siIFI27) for 16 h, followed by transfection of pHBV1.3 for an additional 3 days. HBV RNA was analyzed by Northern blot. The expression of endogenous IFI27 was analyzed by Western blot. β-Actin served as the protein loading control. The relative levels of HBV 3.5 kb RNA in the siIFI27 group were shown as percentages of the siControl group. (**D**) HepG2-NTCP cells were infected with HBV (500 virion genome equivalents [vge]/cell) for 24 h, followed by transfection with control vector or FLAG-IFI27. Cells continued to be cultured for an additional 5 days. The harvested cells were subjected to HBV total RNA Northern blot, HBV core DNA Southern blot, and FLAG-IFI27 Western blot analyses as described above. β-Actin served as the protein loading control. The relative levels of HBV 3.5 kb RNA and ssDNA are presented as percentages of the control groups. The results shown are representative of at least two experimental trials.

To assess the antiviral effect of IFI27 on HBV replication in hepatocyte-derived cells, we co-transfected FLAG-IFI27 or a control vector along with pHBV1.3 into either HepG2 cells or Huh7 cells, followed by analyses of HBV total RNA and cytoplasmic capsid-associated (core) DNA replicative intermediates. As shown in Fig. 2A and B, the overexpression of IFI27 markedly decreased the levels of both HBV core DNA and total RNA, including the 3.5 kb precore mRNA and pgRNA, the latter being the template for HBV reverse transcription (upper panels). Moreover, the quantitative analysis revealed a proportional ~50% reduction in both 3.5 kb RNA and core DNA (lower panels), indicating that IFI27 inhibits HBV DNA replication primarily through reducing viral pgRNA. Given the basal expression of IFI27 detected in both HepG2 and PHH cells (Fig. 1B and C), we next examined whether endogenously expressed IFI27 functions as a restriction factor against HBV transcription. To this end, pHBV1.3 was transfected into HepG2 cells with or without small interfering RNA (siRNA) knockdown (KD) of IFI27. The results revealed that knockdown of basal IFI27 resulted in a modest increase in HBV RNA levels (Fig. 2C), suggesting that IFI27 functions as an endogenous restriction factor for HBV under basal conditions.

Next, we assessed the antiviral effect of IFI27 in HBV-infected HepG2-NTCP cells. Upon HBV infection, the overexpressed IFI27 markedly reduced the levels of HBV DNA and 3.5 kb viral RNA levels as revealed by Southern and Northern blots, respectively (Fig. 2D). Interestingly, Southern blot analysis showed that only the ssDNA intermediate, but not the rcDNA, was reduced by IFI27, suggesting that the majority of rcDNA in HBV-infected HepG2-NTCP cells is derived from the inoculated rcDNA in HBV virions, while the ssDNA indicates *de novo* HBV DNA replication, which is consistent with our previous studies (35–37).

**Fig 3:**
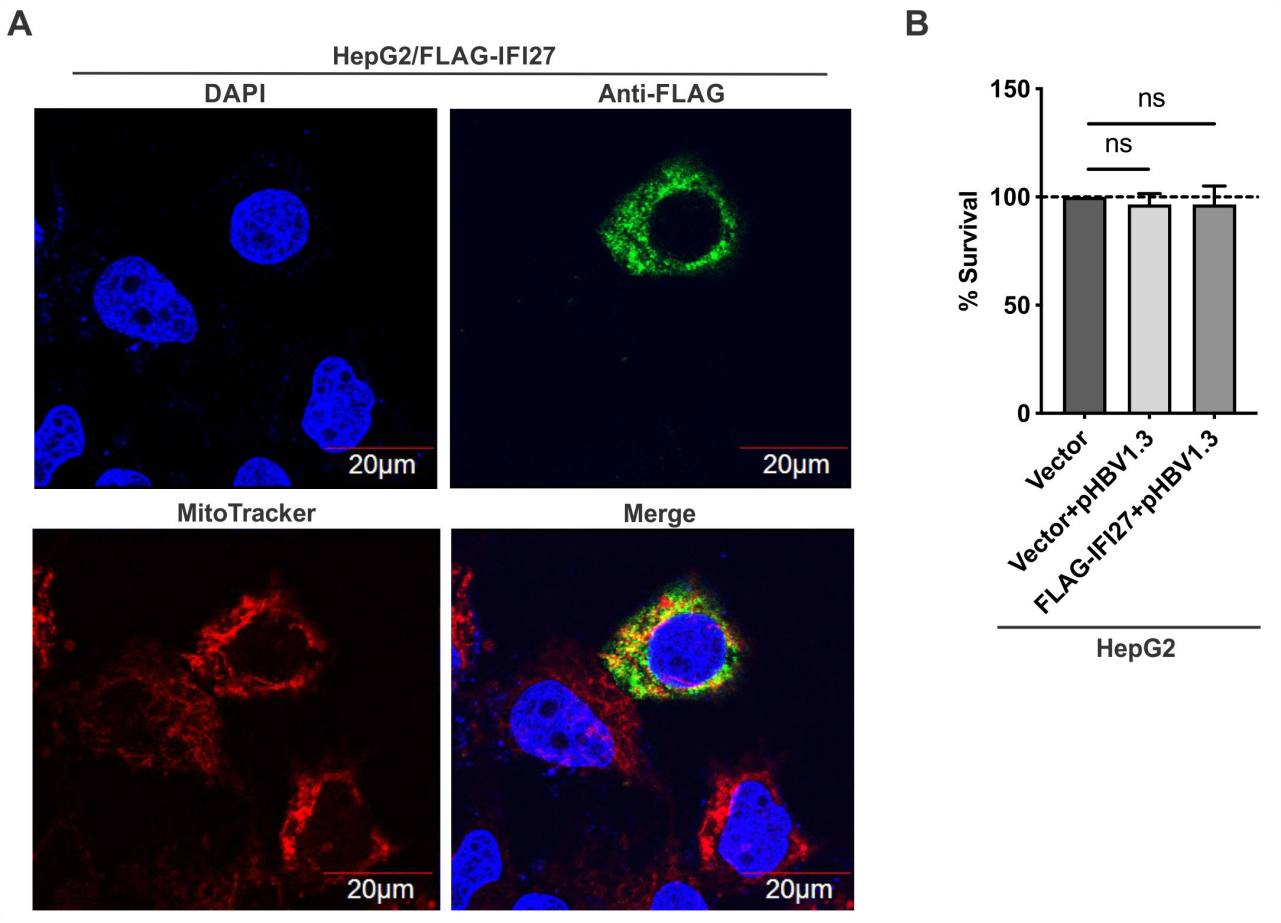
Subcellular localization of ectopically expressed IFI27 and its effect on cell viability. (**A**) HepG2 cells were transfected with FLAG-IFI27 for 3 days, and the subcellular localization of FLAG-IFI27 was detected by immunofluorescence (green) using anti-FLAG antibodies. The cell nuclei were stained with 4´,6-diamidino-2-phenylindole (DAPI) (blue), and mitochondria were stained with MitoTracker (red). (**B**) HepG2 cells were transfected with control vector, control vector plus pHBV1.3, or pHBV1.3 plus FLAG-IFI27 for 4 days, followed by cell viability measurement by the MTT assay. The relative cell survival rate was normalized to the control vector group (mean ± SD, *n* = 3; ns: not significant).

Previous studies have shown that IFI27/ISG12a can sensitize cytokine- or DNA damage-induced cell apoptosis through the formation of pores, inducing permeability in the inner mitochondrial membrane and disrupting the mitochondrial membrane potential (38, 39). Consistent with the reported mitochondrial localization of IFI27, our confocal immunofluorescence assay confirmed that the ectopically expressed FLAG-IFI27 was predominantly and widely localized in cytoplasm, including the perinuclear areas of HepG2 cells, and it was largely associated with mitochondria in the cytoplasm, as evidenced by the colocalization of FLAG-IFI27 and MitoTracker (Fig. 3A). Therefore, we then examined whether the observed anti-HBV effects of IFI27 are related to cell death. We transfected HepG2 cells with either a control vector, pHBV1.3 plus control vector, or pHBV1.3 plus FLAG-IFI27. As anticipated, HBV transfection did not reduce cell viability in the 3-(4,5-dimethylthiazol-2-yl)-2,5-diphenyltetrazolium bromide (MTT) assay due to the noncytopathic nature of HBV replication (40), and the cell viability remained unchanged in cells transfected with both HBV and FLAG-IFI27, suggesting that the observed anti-HBV effect of IFI27 does not involve cytotoxicity (Fig. 3B).

**Fig 4:**
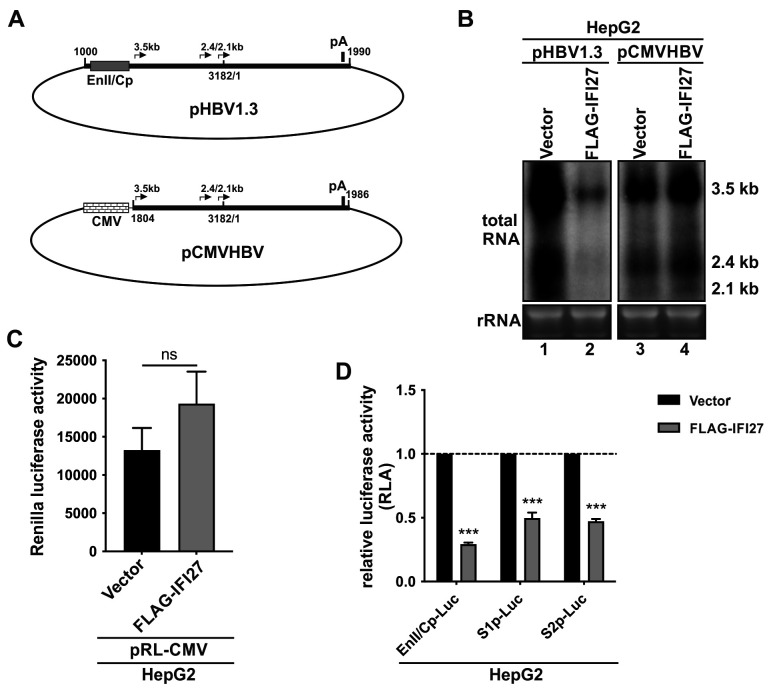
IFI27 overexpression inhibits HBV promoter activity. (**A**) Schematic representation of HBV plasmid pHBV1.3 and pCMVHBV. The promoters controlling pgRNA transcription, specifically the HBV EnII/Cp and CMV-IE promoter, are denoted. The nucleotide (nt) positions corresponding to HBV genome (genotype D) at the start and end of the HBV sequence on the plasmids are shown; nt 3182/1 indicates the EcoRI site. The transcription initiation sites of HBV 3.5 kb RNA and 2.4/2.1 kb surface mRNAs are marked; pA indicates the polyadenylation site for all HBV mRNAs. (**B**) HepG2 cells were co-transfected with pHBV1.3 or pCMVHBV plus either a control vector or plasmid FLAG-IFI27 for 3 days, followed by Northern blot analysis of HBV RNA. (**C, D**) HepG2 cells in 96-well plates were co-transfected with 100 ng of each indicated HBV promoter reporter plasmid (EnII/Cp-Luc, S1p-Luc, S2p-Luc), 4 ng of pRL-CMV, plus 100 ng of control vector or FLAG-IFI27, for 3 days, followed by (**C**) *Renilla* and (**D**) firefly luciferase activity analyses. The *Renilla* luciferase activity readouts were compared between vector and FLAG-IFI27 groups (**C**). The plotted relative firefly luciferase activities (RLA) of the indicated HBV promoters represent the ratio of luminescence from wells expressing FLAG-IFI27 to that from control vector-transfected wells, normalized to *Renilla* luciferase as an internal reference (**D**). Data in the histograms are shown as mean ± SD (*n* = 3); ***P < 0.001; ns, not significant.

Given that IFI27 inhibits HBV replication primarily by reducing the steady-state level of HBV RNA, this process could be potentially attributed to either a transcriptional or post-transcriptional mechanism. To this end, we first compared the antiviral effect of IFI27 between two HBV replicons, pHBV1.3 and pCMVHBV, in which the transcription of pgRNA is governed by HBV core promoter and human cytomegalovirus immediate-early (CMV-IE) promoter, respectively (41) (Fig. 4A). As shown in Fig. 4B, IFI27 overexpression only resulted in a significant reduction of HBV RNA transcribed from pHBV1.3 but not pCMVHBV, indicating a viral promoter-specific antiviral activity of IFI27 and an absence of IFI27-mediated post-transcriptional HBV RNA destabilization. Next, to evaluate the effect of IFI27 on HBV promoter activities, a cell-based luciferase (Luc) reporter assay was conducted. The results revealed that IFI27 overexpression did not reduce the *Renilla* luciferase (RL) signal controlled by CMV-IE promoter on plasmid pRL-CMV (Fig. 4C), which is consistent with the pCMVHBV result (Fig. 4B). By using pRL-CMV as a control to normalize transfection efficiency, IFI27 exhibited significant suppression of the activity of HBV promoters tested, including the Enhancer II and Core promoter (EnII/Cp), S1 promoter (S1p), and S2 promoter (S2p) (Fig. 4D). Collectively, the above results suggest that IFI27 reduces HBV RNA transcription by inhibiting viral promoter activity.

### Assessment of the antiviral effect of endogenous IFI27 on HBV infection

**Fig 5:**
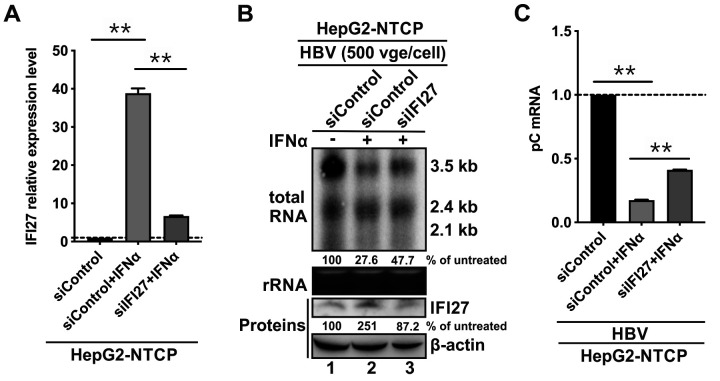
The endogenous IFI27 plays a role in IFNα-elicited antiviral effect in the HBV infection system. HepG2-NTCP cells were transfected with 20 nM of control siRNA (siControl) or IFI27 siRNA (siIFI27) for 16 h, followed by HBV infection at 500 virion genome equivalents [vge]/cell for 24 h, then the infected cells were left untreated or treated with 1,000 IU/mL of IFN-α for an additional 6 days. The cells were harvested and subjected to the following analyses: (**A**) The IFI27 mRNA knockdown efficiency was verified by RT-qPCR. The relative levels of IFI27 mRNA with or without IFNα treatment are plotted as fold change to the siControl group. (**B**) The IFI27 protein was analyzed by Western blot, and β-Actin served as the protein loading control. HBV total RNA was detected by Northern blot. The relative levels of HBV 3.5 kb mRNA and IFI27 are shown as percentages of the untreated siControl groups. (**C**) HBV pC mRNA was detected by RT-qPCR. The relative HBV pC mRNA levels are presented as fold change compared with the untreated siControl group (mean ± SD, *n* = 3. **P < 0.01).

Given that IFI27 has a detectable basal level of expression in hepatocytes, and it can be further induced by IFNα (Fig. 1), it is of interest to assess the role of endogenous IFI27 in IFNα-elicited antiviral response in the context of HBV infection. Hence, we performed IFI27 KD by siRNA in HepG2-NTCP cells. The IFI27 KD cells and control KD cells were then infected by HBV and treated with or without IFNα. As shown in Fig. 5A, IFNα treatment significantly upregulated IFI27 at the mRNA level in control KD cells, but the induction of IFI27 by IFNα was dramatically abolished upon IFI27 knockdown. Next, the outcomes of HBV infection were analyzed. The Northern blot and qPCR analyses demonstrated that IFNα profoundly inhibited HBV infection by reducing viral RNA levels in control KD cells; however, knockdown of IFI27 to 87.2% of its basal level partially, but markedly, abrogated the antiviral activity of IFNα in HBV-infected cells (Fig. 5B and C). The data above suggest that IFI27 functions as a host restriction factor and contributes to IFNα-mediated anti-HBV response, alongside many other antiviral ISGs known to reduce HBV RNA, including ZAP, ISG20, MX2, PML, etc. (20, 22, 24, 25, 42).

### IFI27 inhibits HBV transcription without drastically altering the host transcriptome

**Fig 6:**
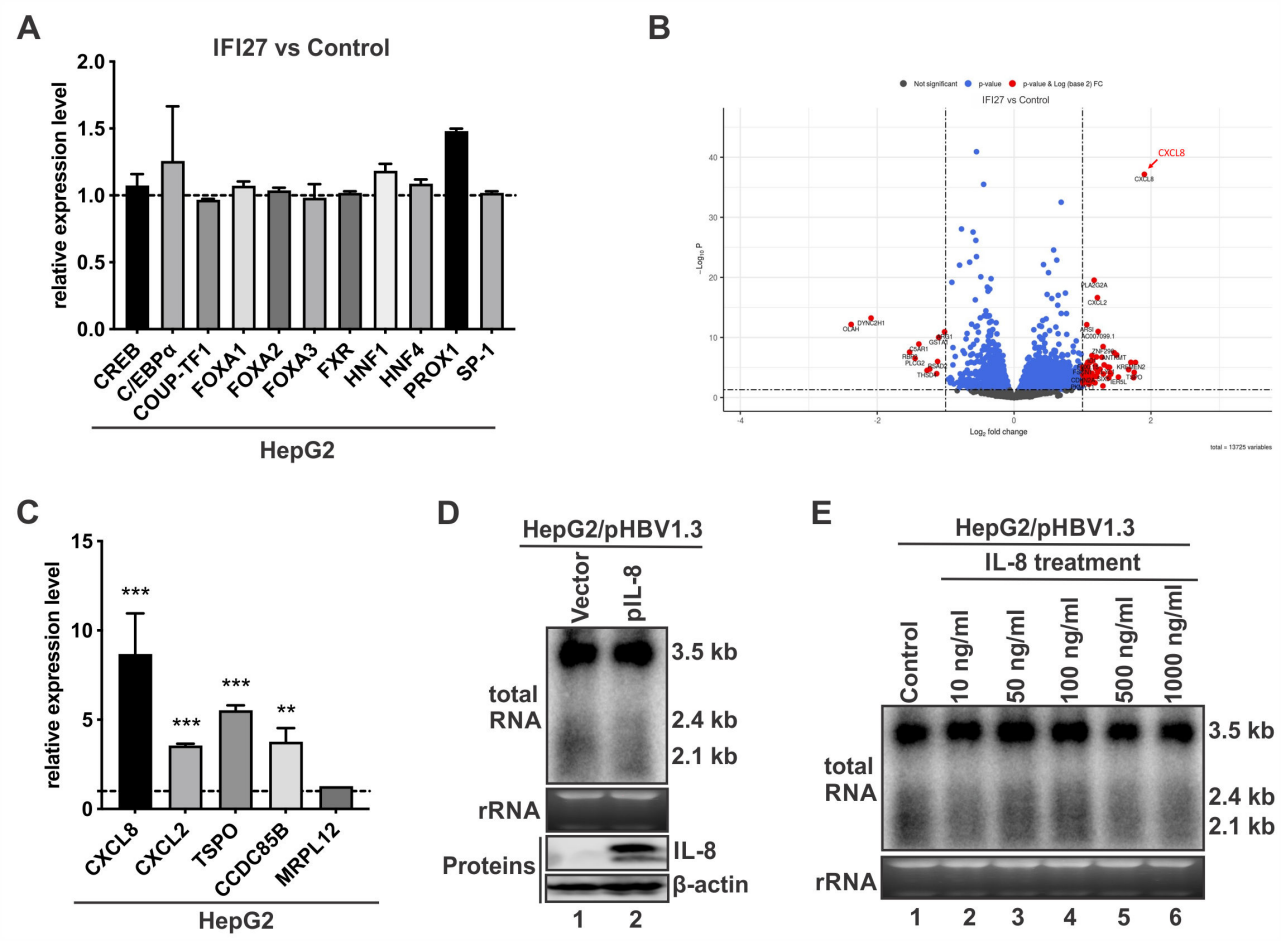
Assessment of the effect of IFI27 on cellular gene transcription. (**A**) Assessing the effect of IFI27 on mRNA levels of selected cellular transcription factors. HepG2 cells were transfected with the control vector or FLAG-IFI27 for 4 days. The mRNA levels of indicated transcription factors were detected using qPCR with specific primers (Table S1). The relative mRNA levels of each transcription factor in the FLAG-IFI27 overexpression group are plotted as fold changes compared with the control vector group, following normalization to β-actin mRNA as an internal reference (mean ± SD [*n* = 3]). (**B**) HepG2 cells were transfected with either a control vector or FLAG-tagged IFI27 for 4 days, followed by mRNA sequencing and differential gene expression analysis. The volcano plot illustrates the distribution of genes based on log_2_ fold change (*x*-axis) and –log_10_ (*P*-value) (*y*-axis), representing both the magnitude and statistical significance of expression changes between the comparison. Each point corresponds to a single gene. Red points denote significantly differentially expressed genes (DEGs) with *P*-values <0.1 and absolute log_2_ fold changes >1. Blue points represent genes with *P*-values <0.1 but log_2_ fold changes between –1 and 1. Black points indicate non-significant genes (*P*-value ≥0.1). The CXCL8 (interleukin-8 [IL-8]) gene is indicated with an arrow. (**C**) The selected DEGs from (**B**) were validated by RT-qPCR using gene-specific primers (Table S1). After normalization to β-actin mRNA, the relative DEG mRNA levels in FLAG-IFI27-transfected HepG2 cells are plotted as fold changes versus the control vector group (mean ± SD [*n* = 3]; **P < 0.01, ***P < 0.001). (**D**) Overexpression of IL-8 does not inhibit HBV transcription. HepG2 cells were co-transfected with pHBV1.3 and either a control vector or plasmid pIL-8 expressing human IL-8 for 4 days, followed by HBV RNA Northern blot and IL-8 Western blot analyses. β-Actin served as the protein loading control. (**E**) IL-8 treatment has no effect on HBV transcription. HepG2 cells transfected with pHBV1.3 were either left untreated or treated with recombinant human IL-8 at the indicated concentrations, and the treatment was refreshed every other day over a 4 day period. The cells were then harvested for HBV RNA Northern blot analysis.

HBV transcription relies on both the ubiquitous and liver-enriched TFs, including SP-1, CREB, C/EBPα, HNF4α, HNF1, FXR, etc. (43–45). To investigate the antiviral mechanism of IFI27 against HBV transcription, we first examined the effect of IFI27 on a list of TFs required for HBV transcription. The results showed no significant changes in the mRNA levels of tested TFs required for HBV transcription after IFI27 overexpression (Fig. 6A).

Next, to identify potential host factors/pathways involved in IFI27-mediated anti-HBV effect, we conducted comparative transcriptomic analysis between HepG2 cells with and without IFI27 overexpression. As depicted in Fig. 6B, IFI27 did not induce a significant change in the host gene transcriptome, and only a few genes showed a fold change greater than 1×log_2_. Among them, the gene with the highest level of upregulation was CXCL8, encoding interleukin-8 (IL-8). To validate the expression of the most upregulated genes in the RNA-seq results, an RT-qPCR analysis was performed (Fig. 6C). As observed, the CXCL8 gene was upregulated by approximately eightfold in HepG2 cells under IFI27 overexpression; other genes of interest, including CXCL2, TSPO, and CCDC85B, also exhibited varying degrees of upregulation. Considering that CXCL8 is the most prominently upregulated gene, it is worth investigating its role in the IFI27-mediated anti-HBV effect. We first tested whether IL-8 could inhibit HBV replication. As shown in Fig. 6D and E, neither intracellular overexpression of IL-8 nor addition of IL-8 to the cell culture supernatant inhibited HBV transcription in pHBV1.3-transfected cells.

It has been reported that the IL-8 receptors CXCR1 and CXCR2 are highly expressed in HepG2, Huh7, and other liver cancer cells (46). Moreover, IL-8 stimulation has been shown to activate a positive feedback loop, increasing the expression of IL-8 itself as well as CXCR1 and CXCR2, and to induce downstream factors such as N-cadherin, E-cadherin, and CD97 (47–49). To assess the functional responsiveness of HepG2 cells to IL-8, we treated the cells with IL-8 and examined the expression of IL-8, CXCR1, CXCR2, N-cadherin, E-cadherin, and CD97. As shown in Fig. S1, IL-8 treatment resulted in significant upregulation of all these genes, confirming that the IL-8 signaling pathway is active in HepG2 cells. Nonetheless, IL-8 treatment did not affect HBV transcription in HepG2 cells (Fig. 6D and E), supporting a conclusion that IL-8 does not mediate the antiviral effect of IFI27 against HBV.

### IFI27 inhibits HBV replication by promoting a decrease in C/EBPα expression

**Fig 7:**
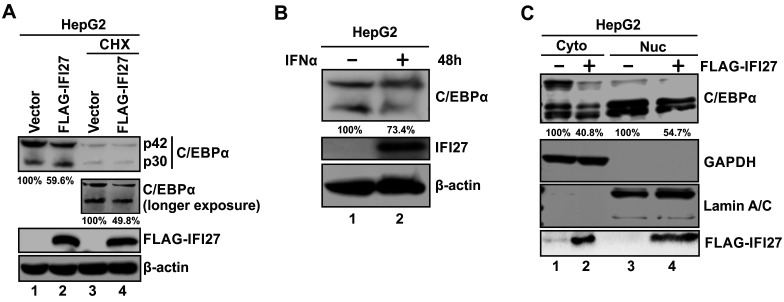
IFI27 reduces the steady-state level of C/EBPα protein. (**A**) IFI27 promotes the protein degradation of C/EBPα. HepG2 cells were co-transfected with control vector or FLAG-IFI27 for 48 h, and then the cells were either left untreated or treated with CHX overnight. The cells were then harvested for protein extraction and Western blot analysis of C/EBPα and FLAG-IFI27. The relative levels of total C/EBPα were quantified and are indicated below the blot. β-Actin served as the protein loading control. (**B**) IFNα induces IFI27 expression and reduces the level of C/EBPα protein. HepG2 cells were left untreated or treated with 1,000 IU/mL of IFNα for 48 h, followed by Western blot analysis of IFI27 and C/EBPα. β-Actin served as the protein loading control. The relative level of total C/EBPα in the IFNα-treated group is shown as a percentage of the untreated group.(**C**) IFI27 primarily induces C/EBPα degradation in the cytoplasm. HepG2 cells were transfected with either a control vector or FLAG-IFI27 for 3 days, followed by cell fractionation and Western blot analyses of FLAG-IFI27 and C/EBPα. GAPDH and Lamin A/C served as a marker and loading control for the cytoplasmic and nuclear fractions, respectively. The relative cytoplasmic (Cyto) and nuclear (Nuc) levels of C/EBPα in the FLAG-IFI27 groups are presented as percentages of their respective control groups.

Given that IFI27 is predominantly localized in the cytoplasm and its overexpression does not cause a significant change in the host transcriptome of HepG2 cells, it is unlikely that IFI27 exerts its anti-HBV transcription effect directly in the nucleus or indirectly through modulating the transcription of TFs required by HBV. Hence, we hypothesized that IFI27 may affect the expression of certain TFs at the protein level. By analyzing the level of several TFs by Western blot, we found that IFI27 overexpression was able to decrease the protein level of C/EBPα. As shown in Fig. 7A, IFI27 overexpression resulted in a reduction of C/EBPα protein in HepG2 cells (lane 2 vs 1), and such effect became more pronounced when new protein synthesis was inhibited by cycloheximide (CHX) (lane 4 vs 3). The upper and lower bands represent the full-length C/EBPα protein of 42 kDa (p42) and the alternative translation product of 30 kDa (p30), respectively, both of which function as transcriptional activators (50). Furthermore, the induction of IFI27 in IFNα-treated HepG2 cells was accompanied by a decrease in C/EBPα, indicating that the IFNα-induced IFI27 is also able to reduce C/EBPα protein (Fig. 7B).

Since IFI27 is predominantly localized in the cytoplasm, we next conducted a cell fractionation experiment to determine the spatial correlation between IFI27 and C/EBPα. As shown in Fig. 7C, IFI27 induced the reduction of C/EBPα primarily in the cytoplasm (top panel, lanes 1 and 2), which consequently led to C/EBPα reduction in the nucleus (top panel, lanes 3 and 4). Interestingly, both the p42 and p30 isoforms displayed a doublet band pattern in the cell fractionation assay, although the underlying reason remains unknown. In addition, while p42 appears to be more abundant than p30 in whole-cell lysate and cytoplasmic fraction, the ratio of these two isoforms becomes reversed in nuclear fraction, indicating differential stability of the two isoforms in the nucleus. While FLAG-IFI27 was detected in the cytoplasmic fraction (bottom panel, lane 2), the detection of FLAG-IFI27 in the nuclear fraction (bottom panel, lane 4) was unexpected as the IF results did not show FLAG-IFI27 within the nucleus (Fig. 3A). We reasoned that the detected nuclear FLAG-IFI27 on Western blot was originally associated with the nuclear membrane at its cytoplasmic side. In this regard, a previous study has found that IFI27 is largely localized to the nuclear membrane in both 293 and HeLa cells (51).

**Fig 8:**
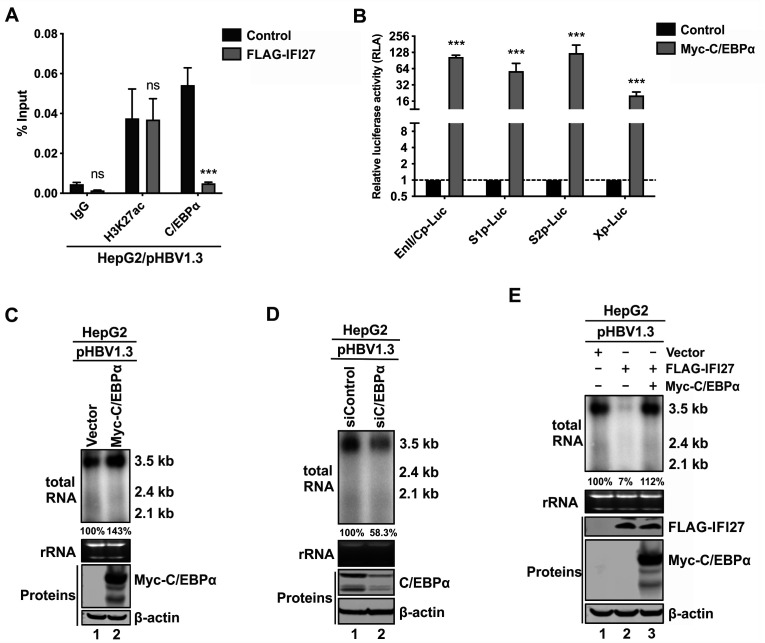
IFI27 suppresses HBV transcription through downregulating C/EBPα. (**A**) IFI27 reduces the enrichment of C/EBPα on HBV genome. HepG2 cells were co-transfected with pHBV1.3 plus either a control vector or FLAG-IFI27 for 3 days. The cells were then subjected to a ChIP assay using ChIP-grade antibodies against H3K27ac (positive control) or C/EBPα, followed by HBV DNA qPCR analysis. The non-immune IgG isotype served as a negative control. The enrichment of IgG, H3K27ac, and C/EBPα on HBV genome is plotted as percentage of input (mean ± SD, *n* = 3. ***P < 0.001, ns, not significant). (**B**) C/EBPα activates HBV promoters. HepG2 cells in 96-well plates were co-transfected with 100 ng each of HBV promoter reporter plasmids (EnII/Cp-Luc, S1p-Luc, S2p-Luc, and X promoter (Xp)-Luc) and 4 ng of pRL-CMV, plus 100 ng of control vector or plasmid expressing Myc-C/EBPα, for 3 days, followed by dual luciferase assay. The relative luciferase activities are plotted as fold changes (mean ± SD, *n* = 3. ***P < 0.001). (**C**) C/EBPα overexpression promotes HBV transcription. HepG2 cells were co-transfected with pHBV1.3 plus either a control vector or plasmid Myc-C/EBPα for 3 days. HBV RNA was analyzed by Northern blot. The expression of Myc-C/EBPα was analyzed by Western blot; β-Actin served as the protein loading control. The relative level of HBV 3.5 kb RNA in the Myc-C/EBPα overexpression group is shown as a percentage of the vector control group. (**D**) Depletion of C/EBPα inhibits HBV transcription. HepG2 cells were transfected with 20 nM of siControl or C/EBPα siRNA (siC/EBPα) for 16 h, followed by transfection with pHBV1.3 for an additional 3 days. The cells were then harvested and subjected to Northern blot analysis of HBV RNA. The relative levels of HBV 3.5 kb RNA are presented as percentages of the siControl group. The knockdown efficiency of C/EBPα was confirmed by Western blot. β-Actin served as the protein loading control. (**E**) C/EBPα overexpression abrogates the IFI27-mediated downregulation of HBV transcription. HepG2 cells were co-transfected with pHBV1.3 plus control vector, FLAG-IFI27, or FLAG-IFI27 and Myc-C/EBPα for 3 days. HBV RNA was analyzed by Northern blot. The expression of FLAG-IFI27 and Myc-C/EBPα was analyzed by Western blot; β-Actin served as the protein loading control. The relative levels of HBV 3.5 kb RNA are shown as percentages of the control group.

It has been reported that C/EBPα binds to HBV Enhancer II, Core promoter, and S2 promoter, which contribute to the transcription of HBV (52–54). As observed in Fig. 8A, the chromatin immunoprecipitation (ChIP)-qPCR assay demonstrated that the binding of C/EBPα to the HBV genome was decreased under IFI27 overexpression, whereas the H3K27ac histone modification on the HBV genome, serving as an internal control, did not show any change. Moreover, using a luciferase assay, we confirmed that C/EBPα overexpression activates all four major HBV promoters (Fig. 8B). Consistently, overexpression of C/EBPα upregulated HBV mRNA levels in pHBV1.3-transfected HepG2 cells (Fig. 8C), and knockdown of endogenous C/EBPα expression in HepG2 cells downregulated the levels of HBV mRNA derived from the pHBV1.3 template (Fig. 8D), confirming the proviral effect of C/EBPα on HBV transcription level.

To assess the effect of IFI27-mediated C/EBPα downregulation in IFI27’s antiviral activity against HBV transcription, we overexpressed C/EBPα in the context of pHBV1.3 and IFI27 co-transfection. As shown in Fig. 8E, IFI27 significantly reduced the HBV RNA level in the absence of C/EBPα (lane 2 vs 1); however, overexpression of C/EBPα significantly abolished the inhibitory effect of IFI27 on HBV RNA (lane 3), indicating that C/EBPα is a critical link between IFI27 and HBV suppression. Collectively, our results demonstrated that IFI27 can induce the reduction of C/EBPα protein and reduce its binding on the HBV genome to prevent it from activating HBV transcription.

### IFI27 recruits SKP2 as an E3 ubiquitin ligase to target C/EBPα for proteasomal degradation

**Fig 9:**
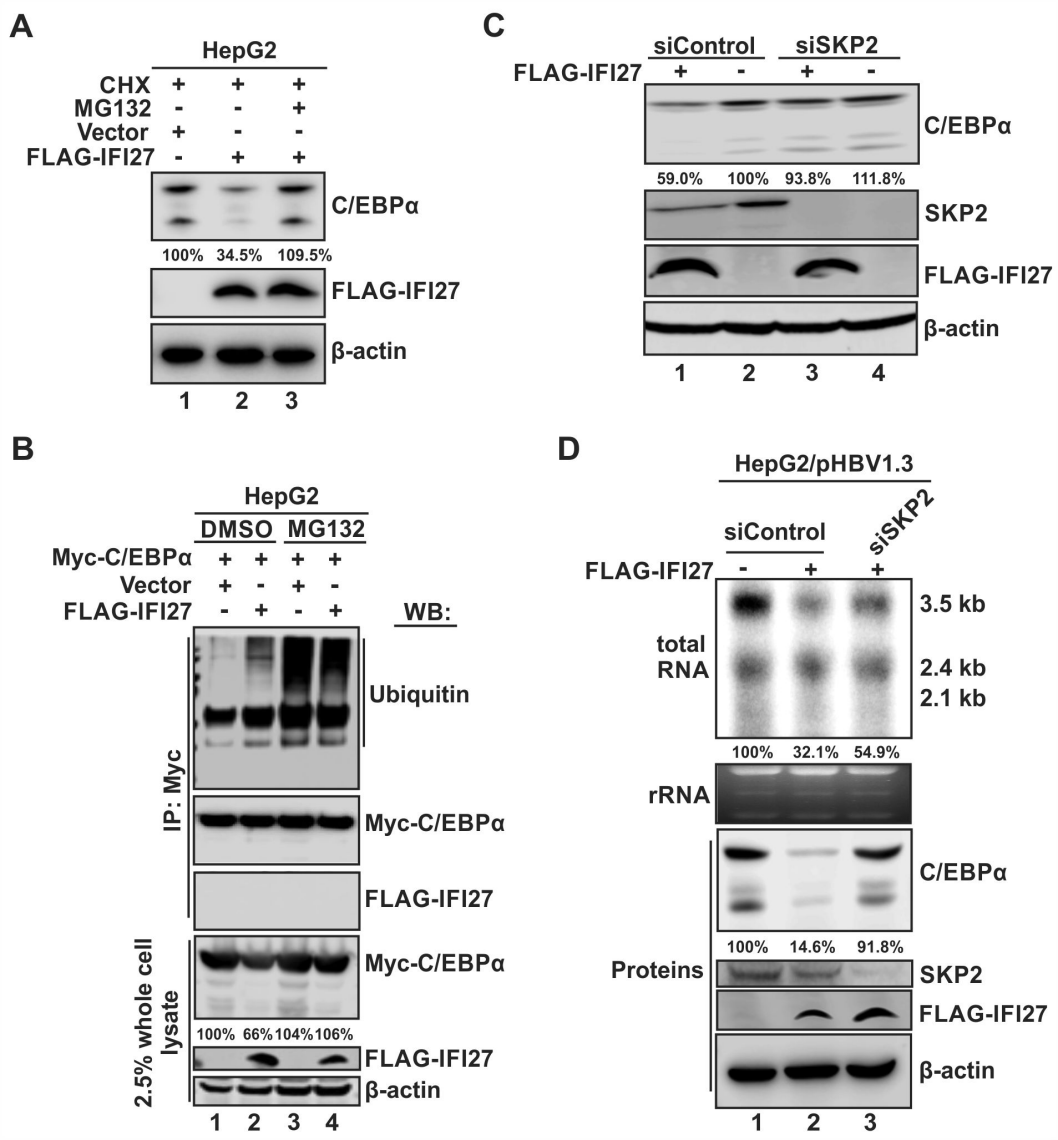
IFI27 downregulates C/EBPα through SKP2-mediated ubiquitin-proteasome degradation. (**A**) The IFI27-mediated C/EBPα degradation can be rescued by proteasome inhibitor MG132. HepG2 cells were transfected with control vector or FLAG-IFI27 for 48 h, followed by treatment with 50 µg/mL of CHX in the absence or presence of 5 µM of MG132, as indicated, for an additional 16 h. Cells were then harvested and subjected to Western blot analysis of C/EBPα and FLAG-IFI27. β-Actin served as a loading control. The relative levels of total C/EBPα are presented as percentages of the vector control group. (**B**) IFI27 promotes C/EBPα ubiquitination. HepG2 cells were transfected with Myc-C/EBPα plus control vector or FLAG-IFI27, as indicated, for 48 h, followed by treatment with dimethyl sulfoxide (DMSO) control or 5 µM of MG132 for another 24 h. Cells were then harvested, and the whole-cell lysates were subjected to immunoprecipitation (IP) using antibody against Myc-tag. The immunoprecipitated samples were analyzed by Western blot (WB) using antibodies against ubiquitin, Myc-tag, and FLAG-tag. A fraction (2.5%) of the whole-cell lysates was analyzed by WB as input controls. The relative levels of Myc-C/EBPα are presented as percentages of the vector control group. β-Actin served as the protein loading control. (**C**) Knockdown of SKP2 blocks IFI27-mediated C/EBPα degradation. HepG2 cells were transfected with 20 nM of siControl or SKP2 siRNA (siSKP2) for 24 h, followed by transfection with FLAG-IFI27 or control vector for an additional 48 h. Cells were harvested and subjected to Western blot detection of C/EBPα, SKP2, FLAG-IFI27, and β-Actin. The relative levels of total C/EBPα are presented as percentages of the siControl plus vector control group. (**D**) Depleting SKP2 partially rescued IFI27-mediated downregulation of HBV transcription. HepG2 cells were transfected with 20 nM of siControl or siSKP2 for 16 h, followed by transfection with pHBV1.3 plus control vector or FLAG-IFI27, as indicated, for another 48 h. Cells were then harvested and subjected to Northern blot analysis of HBV RNA and Western blot analysis of C/EBPα, SKP2, FLAG-IFI27, and β-Actin. The relative levels of HBV 3.5 kb RNA and total C/EBPα are presented as percentages of the siControl groups.

Next, we investigated the mechanism underlying the IFI27-mediated reduction of C/EBPα. By using the proteasome inhibitor MG132, we observed that the IFI27-mediated reduction of C/EBPα was abrogated, indicating that IFI27 promotes C/EBPα degradation via the proteasomal pathway (Fig. 9A). Then, we asked whether IFI27 could induce the ubiquitination of C/EBPα. We conducted a co-immunoprecipitation (co-IP) assay by pulling down Myc-C/EBPα (Fig. 9B). The results revealed an increase in ubiquitination of Myc-C/EBPα when IFI27 was overexpressed (lane 2 vs 1). Moreover, when MG132 was added, the ubiquitination level of Myc-C/EBPα exhibited no difference between the control group and the IFI27 overexpression group (lane 4 vs 3), indicating that the inhibition of proteasome prevented the degradation of ubiquitinated Myc-C/EBPα. These results suggest that IFI27 enhances the ubiquitination of C/EBPα, leading to its degradation through the ubiquitin-proteasome pathway. It is worth noting that the co-IP assay did not reveal an interaction between IFI27 and C/EBPα.

Ubiquitination specifies a stepwise enzymatic cascade that conjugates ubiquitin to target proteins covalently, and the final step requires direct interaction between an E3 ubiquitin ligase and its specific substrate (55). Although IFI27 does not directly interact with C/EBPα, the observed ubiquitination of C/EBPα induced by IFI27 indicates the involvement of an E3 ubiquitin ligase, which is likely recruited by IFI27 to facilitate C/EBPα ubiquitination. In this regard, our literature search identified SKP2, a known E3 ubiquitin ligase, as a potential candidate involved in IFI27-mediated C/EBPα degradation. A previous study demonstrated that IFI27 restricted HCV infection by inducing the ubiquitination and degradation of the viral protein NS5A through promoting SKP2-NS5A interaction (29). Other studies reported that cyclin-dependent kinase 2 triggers the degradation of C/EBPα through SKP2 in acute myeloid leukemia (AML) (56), and that SKP2 ubiquitinates C/EBPα through physically interacting with the C-terminal portion of C/EBPα in AML (57). Therefore, it is of interest to assess the potential role of SKP2 in IFI27-mediated HBV RNA reduction by comparing the inhibitory effect of IFI27 on C/EBPα expression between SKP2 KD and control KD groups. As shown in Fig. 9C, the overexpression of IFI27 consistently resulted in a decrease in the level of C/EBPα; however, when SKP2 was knocked down, the reduction effect was abolished, indicating that SKP2 is responsible for the IFI27-mediated C/EBPα degradation. We next assessed the antiviral activity of IFI27 in HBV-transfected HepG2 cells with and without knockdown of SKP2. As shown in Fig. 9D, depleting SKP2 partially abrogated the IFI27-mediated reduction of HBV RNA. Collectively, the above results revealed that IFI27 degrades C/EBPα through the ubiquitin-proteasome pathway, a process facilitated by the E3 ubiquitin ligase SKP2, thereby hindering C/EBPα binding to the HBV promoter and its activation of HBV transcription.

## DISCUSSION

**Fig 10:**
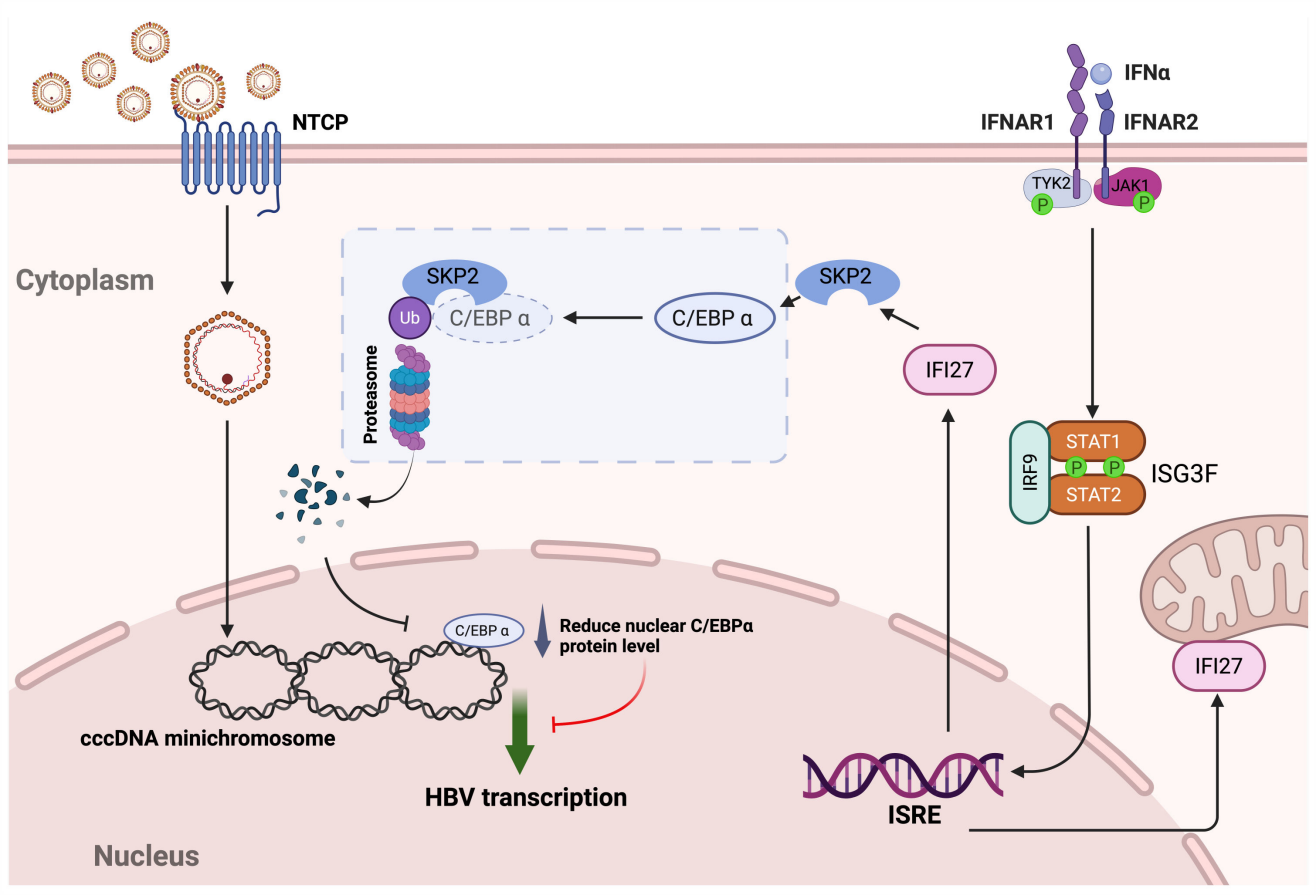
The schematic model of the IFI27-mediated antiviral effect on HBV transcription. HBV infects hepatocytes via NTCP-mediated virus entry, followed by cccDNA minichromosome establishment in the cell nucleus, where the cellular transcription factor C/EBPα binds to HBV cccDNA and promotes viral transcription. Upon IFNα treatment, the engagement of IFNα with the heterodimeric interferon alpha receptor 1/2 (IFNAR1/2) activates the intracellular JAK-STAT signaling pathway, leading to induction of ISGs, including IFI27. The upregulation of IFI27 expression in the cytoplasm facilitates the proteasomal degradation of C/EBPα via recruiting an E3 ubiquitin ligase SKP2 for C/EBPα ubiquitination, thereby leading to the downregulation of nuclear levels of C/EBPα and subsequent HBV transcription. See the text for more details. The model figure was created with BioRender.com.

The innate immune response serves as the first line of defense against numerous kinds of viral invaders, and IFNs play an essential role in the restriction of virus replication and propagation. More than 300 ISGs can be upregulated by interferons, functioning as innate immune effectors in antiviral responses, while some ISGs may also act as proviral factors for certain viruses (16, 58). In terms of HBV infection, it is largely acknowledged that HBV encodes mechanisms to evade innate surveillance, at least in hepatocytes (59, 60). Nonetheless, IFNα-based therapies have been used to treat chronic HBV infection for more than 3 decades, and a better understanding of ISGs’ functions will help identify some essential points that may improve the outcomes of IFNα therapy and may even lead to the development of novel therapeutic approaches (20). In search of ISGs that inhibit HBV replication, we have previously obtained preliminary results showing that IFI27 overexpression inhibited HBV transcription and replication in pHBV1.3-transfected HepG2 cells (26). In the present study, we focused on validating the antiviral effect of IFI27 on HBV transcription and elucidating the underlying antiviral mechanism. We first demonstrated the high inducibility of IFI27 in human hepatocyte cells, including both hepatoma cell lines and PHHs, upon IFNα treatment, as well as the nonapoptotic antiviral activity of IFI27 (Fig. 1 to 3). Furthermore, IFI27 inhibits HBV transcription primarily by downregulating viral promoter activities (Fig. 2, 4, and 5). This effect is at least partially attributed to a mechanism in which IFI27 promotes the degradation of a cellular transcription factor required by HBV, specifically C/EBPα, through the ubiquitin-proteasome degradation pathway (Fig. 6 to 8). Lastly, an E3 ubiquitin ligase SKP2 is responsible for the IFI27-mediated C/EBPα degradation (Fig. 9). Our study thus provides further evidence suggesting that IFI27 is an antiviral ISG against HBV infection, and a model elucidating the IFI27-mediated inhibition of HBV infection by IFNα is proposed for the following discussion (Fig. 10).

Previous studies have reported that IFI27 exerts a broad-spectrum antiviral activity against multiple viruses *in vitro* and/or *in vivo*, including Sindbis virus, HBV, HCV, West Nile virus, Newcastle disease virus, and human immunodeficiency virus 1 (HIV-1), albeit through different mechanisms (26, 29–32, 61). In this study, we further investigated the antiviral effect and mechanism of IFI27 against HBV by utilizing various *in vitro* HBV replication systems, including transfection and infection models in multiple human hepatocyte-derived cell types. It has been previously reported that IFI27 is localized to mitochondria and possesses the pro-apoptotic activity to potentiate cell apoptosis by modulating mitochondrial membrane potential and releasing apoptosis-inducing factors in non-hepatic HT1080 and HEK293 cell lines (38, 39). In our study, we observed that IFI27 is primarily expressed in the cytoplasm and partially co-localized with mitochondria (Fig. 3). However, we did not observe cell viability change upon IFI27 overexpression in HBV-transfected HepG2 cells, inferring a nonapoptotic antiviral activity of IFI27 in the context of HBV replication, although HBV has been reported to possess both pro- and anti-apoptotic activities (62–64). Nonetheless, in our study, IFI27 was demonstrated to be a novel anti-HBV ISG that inhibits HBV transcription via suppressing viral promoters’ activities (Fig. 4).

HBV cccDNA serves as the *bona fide* transcription template for all viral mRNAs, and it resides in host cell nucleus as a stable episome, which is organized into a minichromosome decorated by histones and various viral and cellular non-histone proteins (10). Consistent with the results obtained from HBV and IFI27 transfection systems, the antiviral effect of IFNα-induced endogenous IFI27 on cccDNA transcription was also confirmed in HBV infection system (Fig. 5). The transcription of cccDNA minichromosome is substantially regulated by epigenetic modifications of cccDNA-bound histones, and the HBV-encoded accessory protein HBx plays an indispensable role in supporting cccDNA transcription by maintaining a transcriptionally active epigenetic state of cccDNA (10, 65–69). Additionally, HBc has also been suggested to interact with the cccDNA, although whether it regulates cccDNA transcription remains debatable (65, 70–79). In light of the above information, we tested the possibility that IFI27 may potentially interact with viral factors and consequently influence HBV transcriptional activity. However, IFI27 remained active in inhibiting viral transcription in cells transfected with HBV with HBx-null or HBc-null mutants (data not shown), ruling out the involvement of HBx or HBc in the IFI27-mediated inhibition of HBV transcription. Since IFI27 is primarily localized in the cytoplasm (38, 39) (Fig. 3), and a recent study suggests that it can regulate the AKT-β-catenin-PD-L1 nexus in NK cells (80), we thus speculated that IFI27 may alter certain signaling pathway(s) through initially interacting with the pathway-related factor(s) in the cytoplasm, which leads to a nuclear event that acts on HBV transcription. However, transcriptomic analyses revealed that IFI27 did not significantly alter the host transcriptome in HepG2 cells, including pathways and factors known to regulate HBV transcription (Fig. 6A and B). Moreover, the most highly induced factor, IL-8, showed no antiviral activity against HBV transcription in subsequent functional validation assays (Fig. 6C through E).

Although IFI27 overexpression does not alter the mRNA levels of a list of representative transcription factors known to regulate HBV transcription (Fig. 6A), previous studies demonstrated that IFI27 can directly bind to and promote the degradation of certain viral proteins (29, 61). Inspired by these findings, we hypothesized that IFI27 might impact the protein stability of certain transcription factor(s) required by HBV rather than inhibiting their mRNA expression. Indeed, we found that overexpression of IFI27 resulted in a reduction of C/EBPα at the protein level, and that IFNα treatment also led to a reduction of C/EBPα protein accompanied by an induction of IFI27 (Fig. 7A and B), inferring that C/EBPα might be targeted by IFI27 for inhibition of HBV transcription. Moreover, we observed that IFI27 overexpression mainly reduces C/EBPα protein in the cytoplasm, resulting in a decrease in the total amount of C/EBPα and its binding with the HBV genome in the nucleus (Fig. 7C and 8A). It has been reported that the p42/p30 ratio of C/EBPα plays a critical role in transcriptional regulation of specific target genes, such as DDIT3 in AML cells, with p30 serving as the primary isoform driving DDIT3 transcription (81). However, since IFI27 reduced the levels of both p42 and p30, it remains unclear which isoform is predominantly responsible for modulating HBV transcription, and this question warrants further investigation.

C/EBPα is the first member of a family of six transcription factors: C/EBPα, -β, -γ, -δ, -ε, and -ζ (82, 83). C/EBPs play a crucial role in promoting gene expression by interacting with gene promoters. Once they are bound to the DNA, they can recruit co-activators, which subsequently modify chromatin structure to an open configuration and recruit basal transcription factors (84). It has been demonstrated that the binding of C/EBPα to HBV promoters and EnII plays a crucial role in the transcriptional activation of HBV and promotes its replication (54, 85–87). In line with previous findings, we also observed that C/EBPα overexpression markedly enhanced the activity of HBV promoters and viral pgRNA level, and knockdown of C/EBPα reduced HBV transcription (Fig. 8B through D). Furthermore, transcomplementation of C/EBPα expression abolished IFI27-mediated inhibition of HBV transcription (Fig. 8E), supporting the conclusion that IFI27 inhibits HBV transcription through downregulating C/EBPα.

In the process of exploring the mechanisms of how IFI27 downregulates C/EBPα expression, we found that the inhibitory effect of IFI27 on C/EBPα expression could be almost completely reversed by proteasome inhibitor MG132 treatment, indicating that IFI27 promotes the proteasomal degradation of C/EBPα (Fig. 9A). Moreover, we demonstrated that IFI27 promotes the ubiquitination of C/EBPα, thereby directing it toward proteasomal degradation (Fig. 9B). Previous studies showed that IFI27 could interact with HIV Gag and HCV NS5A to promote protein degradation (29, 61); however, IFI27 did not appear to bind to C/EBPα in the co-IP assay (Fig. 9B), which might be due to a possible transient and dynamic interaction between C/EBPα and IFI27 (a small 12 kDa hydrophobic protein). The ubiquitin-dependent proteasomal protein degradation mechanism involves the coordinated action of E1 ubiquitin-activating enzymes, E2 ubiquitin-conjugating enzymes, and E3 ubiquitin-protein ligases (88, 89). Among these, E3 ubiquitin ligases play a crucial final role by determining substrate specificity and marking target proteins for proteasomal degradation. A previous study has reported that IFI27 recruits E3 ligase SKP2 to degrade HCV NS5A protein (29). In our study, SKP2 knockdown significantly abrogated the inhibitory effects of IFI27 on C/EBPα expression and HBV transcription (Fig. 9C and D), confirming that the antiviral function of IFI27 requires the activity of E3 ligase SKP2. It is worth noting that both HBV and HCV are hepatotropic viruses, and the IFI27-SKP2-mediated protein degradation mechanism may be utilized by host cells to inhibit both, particularly during HBV/HCV co-infection, as HCV is capable of inducing IFNα and IFI27 (59). Notably, while C/EBPα expression was almost fully restored by SKP2 knockdown under IFI27 overexpression, the IFI27-mediated suppression of HBV transcription was only partially reversed (Fig. 9C and D). The underlying reason remains unclear but may be attributed to incomplete SKP2 depletion or the presence of additional SKP2-independent antiviral mechanisms mediated by IFI27. Regarding the latter possibility, a recent study reported that IFI27 binds to the Cp promoter region (nt 1715–1815) of the transfected pHBV1.3 plasmid in HepG2 cells; however, the presented ChIP-PCR data did not rigorously confirm a sequence-specific binding (90). Moreover, IFI27 is predominantly localized in the cytoplasm of HepG2 cells (Fig. 3), suggesting that it is unlikely to directly inhibit HBV transcription within the nucleus. To further elucidate the antiviral mechanisms of IFI27, a proteomic analysis of the IFI27 interactome is warranted and is currently underway in our lab.

It is anticipated that the degradation of C/EBPα by IFI27 might have broader effects on the cellular transcriptome. To further explore the downstream transcriptional programs modulated by IFI27-mediated C/EBPα degradation, we performed gene set enrichment analysis (GSEA) of RNA-seq data from IFI27-overexpressing cells versus controls, using the Molecular Signatures Database (MSigDB). The GSEA results revealed that pathways downstream of C/EBPα, including PPAR signaling, fatty acid metabolism, biosynthesis of unsaturated fatty acids, cholesterol metabolism, complement and coagulation cascades, cholesterol biosynthesis (Medicus reference), and steroid biosynthesis (91–94), were downregulated under IFI27 overexpression (Fig. S2). Interestingly, the TAVOR_CEBPA_TARGETS_DN set, which represents genes normally downregulated by C/EBPα, was upregulated by IFI27 (Fig. S2). Given the well-established roles of C/EBPα and PPAR in regulating adipocyte differentiation and metabolic homeostasis (91, 95), these bioinformatic data indicate that IFI27 suppresses C/EBPα expression and its downstream transcriptional network, particularly pathways related to hepatic and lipid metabolism. However, because most of these pathway-associated differentially expressed genes (DEGs) showed <2-fold changes, they were not highlighted in our initial transcriptomic analysis (Fig. 6B). The relatively mild impact of C/EBPα loss on cellular target gene transcription, compared with its pronounced effect on HBV transcription, may be due to a higher demand of C/EBPα by viral genome to establish infection, at least in a transient period. The broader biological consequences of IFI27-C/EBPα-mediated cellular transcriptomic reprogramming on host functions and HBV infection remain to be determined and await further investigation.

Taken together, the phenotypic and mechanistic characterizations of IFI27-mediated antiviral effect on HBV replication presented in the current study not only shed light on IFI27 biology and virus-host interaction, but also provide insight into the development of novel antiviral strategies. Furthermore, emerging evidence suggests broader roles for IFI27 in inflammatory diseases, autoimmune disorders, and cancers (96). Therefore, it will be of interest to explore the potential involvement of IFI27 in the progression of HBV-associated chronic hepatitis, cirrhosis, and hepatocellular carcinoma in future studies.

## MATERIALS AND METHODS

### Cell cultures

HepG2 and Huh7 cells were maintained in Dulbecco’s modified Eagle’s medium/F-12 with 10% fetal bovine serum (FBS), 100 U/mL penicillin, and 100 µg/mL streptomycin supplemented. The HepG2-NTCP cell line supporting HBV infection was maintained in the same way as HepG2 and Huh7 cells plus additional 8 µg/mL blasticidin (97). Freshly isolated PHH cells were obtained from the Human Liver Tissue and Hepatocyte Research Resource (funded by NIDDK project no. R24DK139775) at The Pittsburgh Liver Research Center (funded by NIDDK grant no. P30DK120531), University of Pittsburgh, and cultured as previously described (98).

### Plasmids, siRNA, cytokines, and compounds

The HBV (genotype D) replication-competent plasmid pHBV1.3 containing a 1.3-mer replicon of viral genome, and pCMVHBV, transcribing HBV pgRNA under the control of a human CMV-IE promoter, were described previously (26, 41, 99). The HBV promoter firefly Luc reporter plasmids, including the EnII/Cp-Luc, S1p-Luc, S2p-Luc, and Xp-Luc, have been described in our previous publications (25, 100). The CMV-IE promoter *Renilla* luciferase reporter plasmid pRL-CMV was purchased from Promega (Cat# E2261). Plasmid FLAG-IFI27 expresses the N-terminally FLAG-tagged IFI27 (26, 101). The human IL-8 expression plasmid (Cat# RC202075) and Myc-tagged C/EBPα expression plasmid (Cat# RC218955) were purchased from OriGene. The transfection of plasmid DNA into cells was conducted by using Lipofectamine 3000 (Cat# L3000150, Thermo Fisher) according to the manufacturer’s manual.

The siRNA for knocking down IFI27 (Cat# sc-105551), C/EBPα (Cat# sc-37047), SKP2 p45 (Cat# sc-74477), and control siRNA-A (Cat# sc-37007) was purchased from Santa Cruz Biotechnology. Lipofectamine RNAiMAX (Cat# 13778100, Thermo Fisher) was used to transfect siRNA into cell cultures.

The recombinant human IL-8/CXCL8 (Cat# 208-IL) and IFNα-2a (Cat# SRP4594) were purchased from R&D Systems and Sigma-Aldrich, respectively. The compound MTT (Cat# M2003), proteasome inhibitor MG132 (Cat# 474790), and protein synthesis inhibitor cycloheximide (Cat# C7698) were purchased from Sigma-Aldrich and dissolved in DMSO to prepare stock solutions per manufacturer’s recommendations.

### Cytotoxicity assay

Cells were transfected with indicated plasmids for 4 days, then MTT compound was added to the culture supernatant at a final concentration of 0.5 mg/mL, and the plate was incubated at 37°C for 4 h. Next, the supernatant was gently removed by vacuum aspiration, and 150 µL DMSO was added to dissolve the reaction product at 37°C for 30 min. The colorimetric absorbance was measured at 570 nm with BioTek Synergy HTX multimode plate reader. The relative cell survival rate was determined by normalizing the OD_570_ values to control groups.

### HBV infection

The infectious HBV particles were harvested from the supernatant of induced HepAD38 cells, and the quantification of virion genome equivalents was performed by following the previously established methods (97). HBV *in vitro* infection of HepG2-NTCP or PHH cells was conducted according to our previous publications (36, 98, 102).

### IFA

HepG2 cells were transfected with control vector or FLAG-IFI27 for 3 days. Cells were incubated with MitoTracker Deep Red FM (Cat# M22426, Invitrogen) for 20 min according to manufacturer’s directions, followed by fixation with 4% paraformaldehyde for 20 min and permeabilization with 0.5% Triton X-100 in phosphate-buffered saline (PBS) for 1 h at room temperature. Cells were then blocked with IFA blocking buffer (10% FBS plus 2% bovine serum albumin in 1× PBS) for 1 h at room temperature and incubated with anti-FLAG M2 antibody (Cat# F3165, Sigma-Aldrich) at 4°C overnight. After washing with 1× PBS and a second blocking for 30 min, cells were stained with Alexa Fluor 488 dye-conjugated goat anti-mouse secondary antibody (Cat# A1101, Invitrogen), and the nuclei were counterstained with 4´,6-diamidino-2-phenylindole for 30 min at room temperature. Both the primary and the secondary antibodies were diluted in IFA blocking buffer. The cells were washed with 1× PBS and subjected to Olympus FV1000 MPE confocal microscopy analysis with the 60× or 20× objective. Images were analyzed using FV10-ASW 3.0 Viewer and ImageJ software.

### Cellular mRNA qPCR

Total cellular RNA was extracted by TRI Reagent (Cat# AM9738, Thermo Fisher) and subjected to reverse transcription to generate cDNA using SuperScript III reverse transcriptase (Cat# 18080093, Thermo Fisher). Real-time qPCR of cDNA was then performed using gene-specific primers of the indicated transcription factors (Table S1) and SYBR Green master mix (Cat# 04707516001, Roche) on the Roche LightCycler 96 system. Primers for E-cadherin (Cat# HP207683) and N-cadherin (Cat# HP205580) were purchased from OriGene. The relative mRNA expression levels of detected genes were normalized to the mRNA levels of β-actin from the same samples.

### HBV DNA and RNA analysis

Intracellular HBV RNA and core (capsid-associated) DNA were extracted and subjected to Northern blot and Southern blot, respectively, as described previously (25, 103). Hybridization signals were exposed to a phosphorimager screen and scanned using the Typhoon FLA-7000 imager (GE Healthcare). HBV pC mRNA-specific qPCR was conducted according to our previous publications (36, 65). HBV pC mRNA qPCR was normalized to cellular GAPDH mRNA qPCR as previously described (65).

### ChIP-qPCR

To assess the enrichment of C/EBPα on HBV genome, ChIP assay was performed using the ChIP-IT Express Chromatin Immunoprecipitation Kit (Cat# 53008, Active Motif) as previously described (36, 104). Briefly, after the prepared chromatin-containing cell lysate was divided into input and ChIP samples, the latter was subjected to immunoprecipitation with protein G magnetic beads coated with nonimmune serum control IgG antibody (Cat# I8765, Sigma-Aldrich) or ChIP-grade specific antibodies, specifically anti-H3K27ac (Cat# ab4729, Abcam) and anti-C/EBPα (Cat# 8178, Cell Signaling). The ChIPed DNA was cleaned up by QIAquick PCR purification kit (Cat# 28106, Qiagen) and subjected to qPCR with HBV-specific primers as previously described (36, 104). The occupancy of the protein of interest on the HBV genome was calculated and expressed as the percentage of input (% input).

### Western blot assay

Cells were rinsed with cold 1× PBS and lysed in 1× Laemmli buffer. Then, cell lysates were subjected to sonication at 20% amplitude with 10 s impulse and 2 s rest for six cycles using EpiShear Probe sonicator (Active Motif), followed by resolution in either Novex 16% Tricine Gel (Cat# EC66952, Thermo Fisher) or Novex 12% Tris-Glycine Gel (Cat# XP00122, Thermo Fisher), depending on the size of the target protein. The proteins were transferred onto the Amersham Protran 0.45 NC nitrocellulose membrane (Cat# 160002, Cytiva). The membrane was blocked with WesternBreeze blocking buffer (Cat# WB7050, Thermo Fisher) and then probed with primary antibodies against the indicated proteins or epitope tags, including β-Actin (Cat# sc-47778, Santa Cruz), GAPDH (Cat# sc-47724, Santa Cruz), SKP2 p45 (Cat# sc-74477, Santa Cruz), Lamin A/C (Cat# sc-376248, Santa Cruz), ubiquitin (Cat# sc-8017, Santa Cruz), C/EBPα (Cat# 8178S, Cell Signaling), FLAG-tag M2 (Cat# F1804, Sigma-Aldrich), Myc-tag (Cat# 2278, Cell Signaling), and IFI27 (51). After the membrane was washed three times with 1× PBS, it was incubated with WesternSure Goat anti-mouse horseradish peroxidase (HRP) (Cat# 926-80010) or anti-Rabbit HRP (Cat# 926-80011) secondary antibody (Li-COR). Detection of the immunoblot signal was performed using enhanced chemiluminescence (ECL) with SuperSignal West Pico PLUS Luminol/Enhancer (Cat# 34580, Thermo Fisher) and visualized using the Li-COR Odyssey system.

### Coimmunoprecipitation assay

HepG2 cells in six-well plates were transfected with the indicated plasmids for 48 h. Cells were then washed with 1× PBS and lysed in 400 µL lysis buffer (1% NP-40, 10% glycerol, 2 mM EDTA, 50 mM Tris-HCl [pH 7.0], and 150 mM NaCl) supplemented with 1× Halt protease inhibitor cocktail (Cat# 87785, Thermo Fisher) and 100 µM phenylmethylsulfonyl fluoride (PMSF) (Cat# 52332, Sigma-Aldrich) per well. The cell lysates were gently rotated at 4°C for 1 h, followed by centrifugation at 12,000 rpm in 4°C for 10 min. A total of 2.5% of the supernatant was saved as input for Western blot detection. The remaining supernatant was incubated with anti-C/EBPα antibody (Cat# 8178, Cell Signaling) by rotation at 4°C for 1 h. Then, 20 µL of protein A/G beads (Cat# sc-2003, Santa Cruz) were rinsed with the cell lysis buffer and added to the immunoprecipitation reaction. The mixture was rotated at 4°C overnight, and the beads were precipitated by spin-down and washed three times with cell lysis buffer. The supernatant was discarded, and the immunoprecipitated pellet was mixed with 1× Laemmli buffer and resolved by SDS-PAGE for Western blot assay as described above.

### Differential transcriptome analysis

Two groups of differential transcriptome analyses were performed in this study. One group was HepG2-NTCP cells with and without IFNα (1,000 IU/mL) treatment; the other group was HepG2 cells with and without FLAG-IFI27 overexpression. Total RNA was extracted from the cell samples and submitted in triplicate for mRNA sequencing at The Center for Medical Genomics at Indiana University School of Medicine or The Health Sciences Sequencing Core at UPMC Children’s Hospital of Pittsburgh. The differential transcriptome analysis was performed following the protocols and bioinformatic pipeline described in our previous study (36).

### GSEA

RNA-seq data from IFI27-overexpressing and control cells were analyzed by GSEA (v4.4.0; Broad Institute, Cambridge, MA) using the C2 curated gene sets (C2-all) collection from the MSigDB (105–107). Genes were ranked according to differential expression, and enrichment statistics, including normalized enrichment score and false discovery rate, were calculated using default parameters.

### Statistical analysis

Data were analyzed using GraphPad Prism 10.0 and expressed as mean ± standard deviation. Student’s *t*-test was used to determine statistical significance (*P*-value <0.05).

## Data Availability

All data are included in the paper or available upon request. The RNA-seq data sets of this study have been deposited in the NCBI BioProject database (https://www.ncbi.nlm.nih.gov/sra/PRJNA1295100).

## SUPPLEMENTAL MATERIAL

Supplemental material jvi.01509-25-s0001.pdf Table S1; Figures S1 and S2.

ASM does not own the copyrights to Supplemental Material that may be linked to, or accessed through, an article. The authors have granted ASM a non-exclusive, world-wide license to publish the Supplemental Material files. Please contact the corresponding author directly for reuse.
