# Supplementary material for: Interferon alpha-inducible protein 27 (IFI27) inhibits hepatitis B virus (HBV) transcription through downregulating cellular transcription factor C/EBPα
Source: J Virol. 2025 Oct 13;99(11):e01509-25. doi: 10.1128/jvi.01509-25 (PMC12646010; doi:10.1128/jvi.01509-25)
Supplement: Supplemental material — Table S1; Figures S1 and S2. [file jvi.01509-25-s0001.pdf]

SUPPLEMENTAL INFORMATION

Table S1. Primers for cellular mRNA qPCR.

Fig. S1. IL-8 signaling pathway is functional in HepG2 cells. HepG2 cells were left untreated or treated with IL-8 at 1,000 ng/ml for 2 days. The mRNA levels of IL-8 responsive genes were detected by RT-qPCR using gene-specific primers (Table S1). The relative mRNA levels of the indicated genes in the IL-8 treatment group were plotted as fold changes compared to the control vector group, following normalization to β-actin mRNA as an internal reference. Data in the histograms are presented as mean ± SD (n=3); ***$p<0.001$.

Fig. S2. GSEA reveals alteration of C/EBPα target genes upon IFI27 expression. (A) Gene set enrichment analysis (GSEA) of the indicated pathways in IFI27-overexpressing versus control cells. Enrichment scores were calculated based on hallmark and curated gene sets, with significantly enriched pathways ranked by normalized enrichment score (NES). Bars represent enrichment scores, with colors indicating levels of statistical significance: orange and light blue denote highly significant enrichment (FDR $\leqslant$ 0.05), while purple and dark blue indicate significant enrichment at FDR $\leqslant$ 0.25.

Supplemental Table 1: Primers for cellular mRNA qPCR.

Name	Sequence (5'→3')	Reference
HNF4α	Forward: CGTCATCGTTGCCAACACAAT<br>Reverse: GGGCCACTCACACATCTGTC	(1, 2)
HNF1α	Forward: TACACCACTCTGGCAGCCACACT<br>Reverse: CGGTGGGTACATTGGTGACAGAAC	(2, 3)
FOXA1	Forward: GTGGCTCCAGGATGTTAGGA<br>Reverse: GAGTAGGCCTCCTGCGTGT	(4)
FOXA2	Forward: GCGACCCCAAGACCTACAG<br>Reverse: GGTTCTGCCGGTAGAAGGG	(2, 5)
FOXA3	Forward: TCATGTAGGAGTTGAGGGGG<br>Reverse: GAAGATGGAGGCCCATGAC	(2)
C/EBPα	Forward: CCAAGAAGTCGGTGGACAAG<br>Reverse: AGGCGGTCATTGTCACTGGT	(2)
PROX1	Forward: TTGACATTGGAGTGAAAAGGACG<br>Reverse: TGCTCAGAACCTTGGGGATTC	(1, 2)
FXR	Forward: GGACAGAACCTGGAAGTGGA<br>Reverse: CTGCATGCTGCTTCACATTT	(6)
COUP-TF1	Forward: GCCTCAAGAAGTGCCTCAAAGT<br>Reverse: CGAGATGTAGCCGGACAGGTAG	(7)
SP1	Forward: CCTCCAGACCATTAACCTCAG<br>Reverse: TCCACCTGCTGTGTCATCAT	(8)
CREB	Forward: ACTGTAACGGTGCCAACTCC<br>Reverse: GAATGGTAGTACCCGGCTGA	(9)
β-actin	Forward: TGGGCATGGGTCAGAAGGAT<br>Reverse: TCCATCACGATGCCAGTGGT	(10)

CXCR1	Forward: CAGATCCACAGATGTGGGAT<br>Reverse: AGCAGCCAAGACAAACAAACTT	(11)
CXCR2	Forward: CTTTTCTACTAGATGCCGC<br>Reverse: AGATGCTGAGACATATGAATTT	(11)
IL-8	Forward: CTTTGTCCATTCCCACTTCTGA<br>Reverse: TCCCTAACGGTTGCCTTTGTAT	(11)
CD97	Forward: GATACTGCTGGTTGGACTTTGAG<br>Reverse: CCCTCGCCTTCTTTAATTTCTTCA	(12)
E-Cadherin	Forward: GCCTCCTGAAAAGAGAGTGGAAG<br>Reverse: TGGCAGTGTCTCTCCAAATCCG	Origene, Cat# HP207683
N-Cadherin	Forward: CCTCCAGAGTTTACTGCCATGAC<br>Reverse: GTAGGATCTCCGCCACTGATTC	Origene, Cat# HP205580

References

1. Inui J, Ueyama-Toba Y, Mitani S, Mizuguchi H. 2023. Development of a method of passaging and freezing human iPS cell-derived hepatocytes to improve their functions. PLoS One 18:e0285783.
2. Nakamori D, Akamine H, Takayama K, Sakurai F, Mizuguchi H. 2017. Direct conversion of human fibroblasts into hepatocyte-like cells by ATF5, PROX1, FOXA2, FOXA3, and HNF4A transduction. Sci Rep 7:16675.
3. Yan Q, Deng L, Zhao X, Ye L, Fang Y, Meng Y, Wang Z, Luo X, Liu S, Li A. 2018. Establishment and characterization of an immortalized human hepatocyte line for the development of bioartificial liver system. Cytotechnology 70:665-674.
4. Krause WC, Shafi AA, Nakka M, Weigel NL. 2014. Androgen receptor and its splice variant, AR-V7, differentially regulate FOXA1 sensitive genes in LNCaP prostate cancer cells. Int J Biochem Cell Biol 54:49-59.
5. Caron J, Pene V, Tolosa L, Villaret M, Luce E, Fourrier A, Heslan JM, Saheb S, Bruckert E, Gomez-Lechon MJ, Nguyen TH, Rosenberg AR, Weber A, Dubart-Kupperschmitt A. 2019. Low-density lipoprotein receptor-deficient hepatocytes differentiated from induced pluripotent stem cells allow familial hypercholesterolemia modeling, CRISPR/Cas-mediated genetic correction, and productive hepatitis C virus infection. Stem Cell Res Ther 10:221.
6. Bungard CI, McGivan JD. 2005. Identification of the promoter elements involved in the stimulation of ASCT2 expression by glutamine availability in HepG2 cells and the probable involvement of FXR/RXR dimers. Arch Biochem Biophys 443:53-9.

7. Perets R, Kaplan T, Stein I, Hidas G, Tayeb S, Avraham E, Ben-Neriah Y, Simon I, Pikarsky E. 2012. Genome-wide analysis of androgen receptor targets reveals COUP-TF1 as a novel player in human prostate cancer. PLoS One 7:e46467.
8. Citron BA, Dennis JS, Zeitlin RS, Echeverria V. 2008. Transcription factor Sp1 dysregulation in Alzheimer's disease. J Neurosci Res 86:2499-504.
9. Hong JS, Kim SW, Koo JS. 2008. Sp1 up-regulates cAMP-response-element-binding protein expression during retinoic acid-induced mucous differentiation of normal human bronchial epithelial cells. Biochem J 410:49-61.
10. Xie Z, Shen S, Huang K, Wang W, Liu Z, Zhang H, Lu M, Sun J, Hou J, Liu H, Guo H, Zhang X. 2023. Mitochondrial HIGD1A inhibits hepatitis B virus transcription and replication through the cellular PNKD-NF-kappaB-NR2F1 nexus. J Med Virol 95:e28749.
11. Bi H, Zhang Y, Wang S, Fang W, He W, Yin L, Xue Y, Cheng Z, Yang M, Shen J. 2019. Interleukin-8 promotes cell migration via CXCR1 and CXCR2 in liver cancer. Oncol Lett 18:4176-4184.
12. Meng ZW, Zhang L, Cai XR, Wang X, She FF, Chen YL. 2023. IL-8 is a novel prometastatic chemokine in intrahepatic cholangiocarcinoma that induces CXCR2-PI3K/AKT signaling upon CD97 activation. Sci Rep 13:18711.

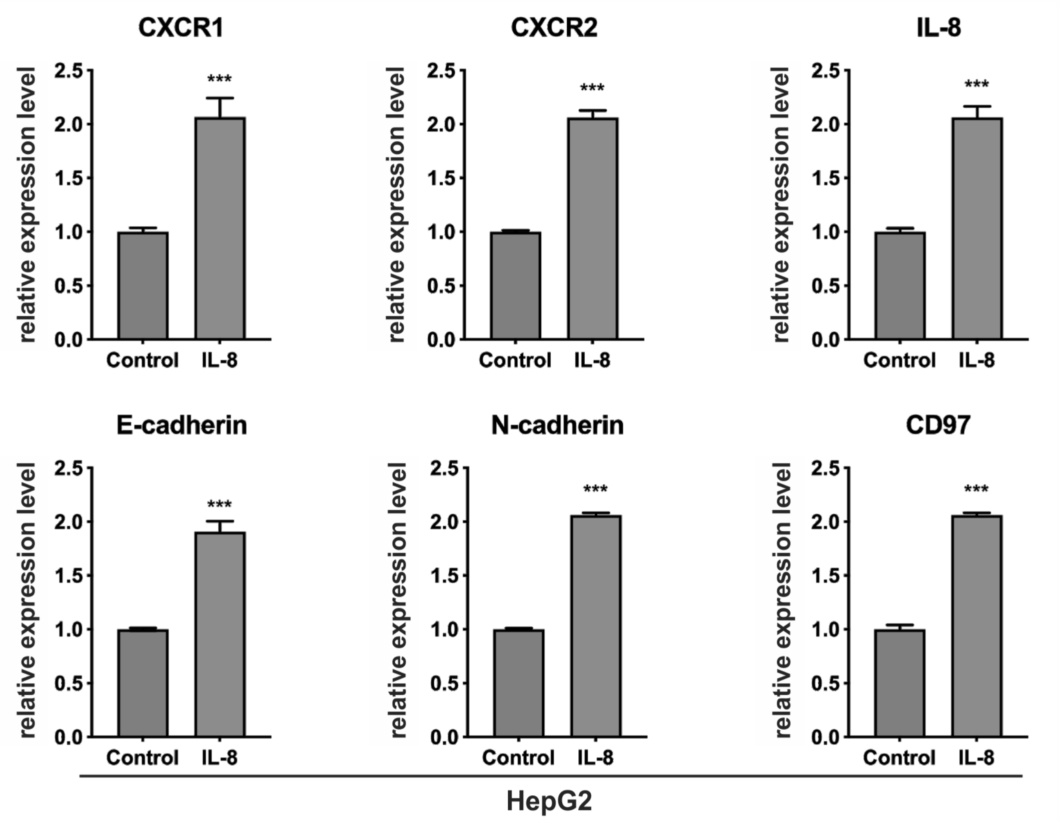

CXCR1
CXCR2
IL-8
E-cadherin
N-cadherin
CD97
relative expression level
2.5
2.0
1.5
1.0
0.5
0.0
***
Control
IL-8
HepG2

Figure S1

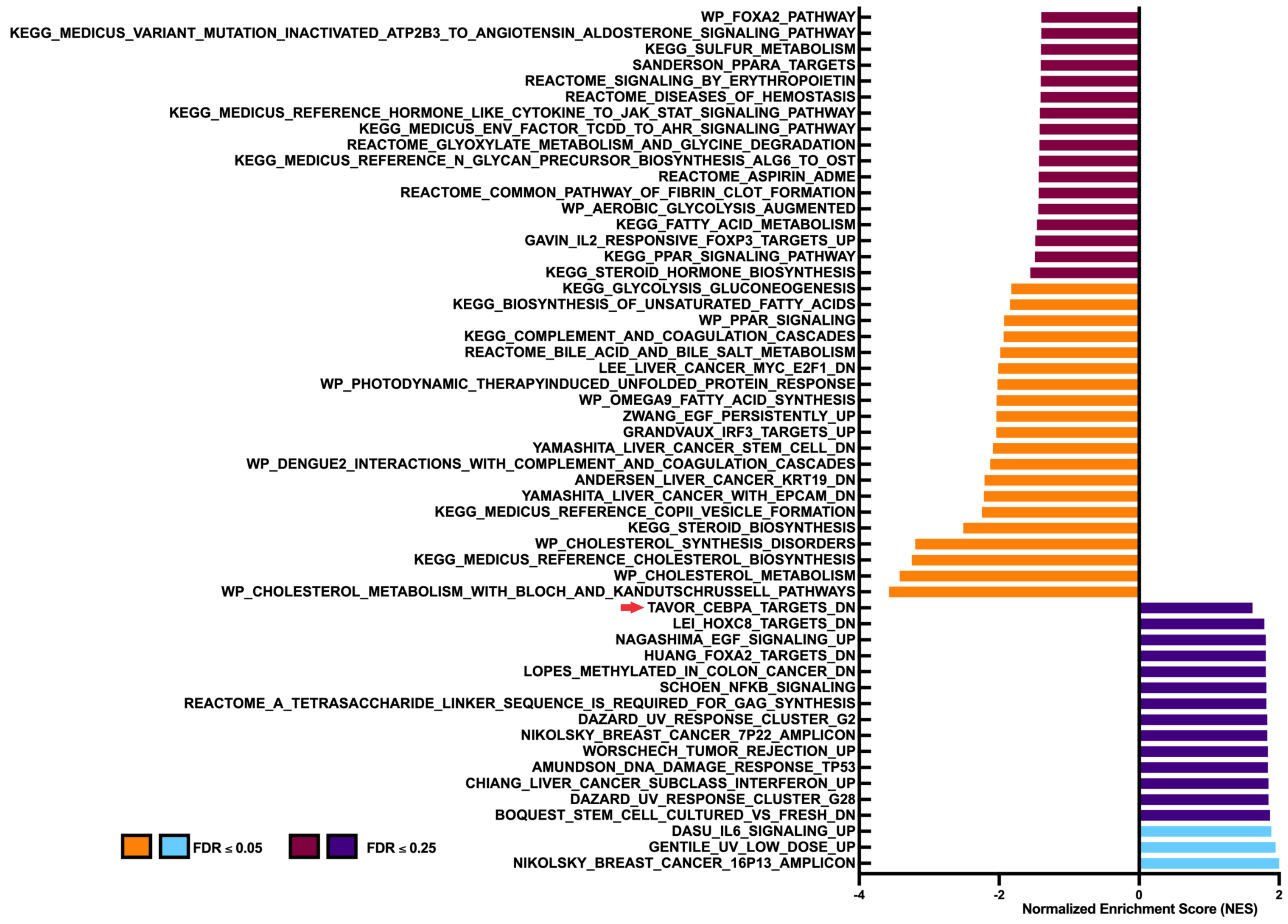
WP_FOXA2_PATHWAY
KEGG_MEDICUS_VARIANT_MUTATION_INACTIVATED_ATP2B3_TO_ANGIOTENSIN_ALDOSTERONE_SIGNALING_PATHWAY
KEGG_SULFUR_METABOLISM
SANDERSON_PPARA_TARGETS
REACTOME_SIGNALING_BY_ERYTHROPOIETIN
REACTOME_DISEASES_OF_HEMOSTASIS
KEGG_MEDICUS_REFERENCE_HORMONE_LIKE_CYTOKINE_TO_JAK_STAT_SIGNALING_PATHWAY
KEGG_MEDICUS_ENV_FACTOR_TCDD_TO_AHR_SIGNALING_PATHWAY
REACTOME_GLYOXYLATE_METABOLISM_AND_GLYCINE_DEGRADATION
KEGG_MEDICUS_REFERENCE_N_GLYCAN_PRECURSOR_BIOSYNTHESIS_ALG6_TO_OST
REACTOME_ASPIRIN_ADME
REACTOME_COMMON_PATHWAY_OF_FIBRIN_CLOT_FORMATION
WP_AEROBIC_GLYCOLYSIS_AUGMENTED
KEGG_FATTY_ACID_METABOLISM
GAVIN_IL2_RESPONSIVE_FOXP3_TARGETS_UP
KEGG_PPAR_SIGNALING_PATHWAY
KEGG_STEROID_HORMONE_BIOSYNTHESIS
KEGG_GLYCOLYSIS_GLUCONEOGENESIS
KEGG_BIOSYNTHESIS_OF_UNSATURATED_FATTY_ACIDS
WP_PPAR_SIGNALING
KEGG_COMPLEMENT_AND_COAGULATION_CASCADES
REACTOME_BILE_ACID_AND_BILE_SALT_METABOLISM
LEE_LIVER_CANCER_MYC_E2F1_DN
WP_PHOTODYNAMIC_THERAPYINDUCED_UNFOLDED_PROTEIN_RESPONSE
WP_OMEGA9_FATTY_ACID_SYNTHESIS
ZWANG_EGF_PERSISTENTLY_UP
GRANDVAUX_IRF3_TARGETS_UP
YAMASHITA_LIVER_CANCER_STEM_CELL_DN
WP_DENGUE2_INTERACTIONS_WITH_COMPLEMENT_AND_COAGULATION_CASCADES
ANDERSEN_LIVER_CANCER_KRT19_DN
YAMASHITA_LIVER_CANCER_WITH_EPCAM_DN
KEGG_MEDICUS_REFERENCE_COPII_VESICLE_FORMATION
KEGG_STEROID_BIOSYNTHESIS
WP_CHOLESTEROL_SYNTHESIS_DISORDERS
KEGG_MEDICUS_REFERENCE_CHOLESTEROL_BIOSYNTHESIS
WP_CHOLESTEROL_METABOLISM
WP_CHOLESTEROL_METABOLISM_WITH_BLOCH_AND_KANDUTSCHRUSSELL_PATHWAYS
TAVOR_CEBPA_TARGETS_DN
LEI_HOXC8_TARGETS_DN
NAGASHIMA_EGF_SIGNALING_UP
HUANG_FOXA2_TARGETS_DN
LOPES_METHYLATED_IN_COLON_CANCER_DN
SCHOEN_NFKB_SIGNALING
REACTOME_A_TETRASACCHARIDE_LINKER_SEQUENCE_IS_REQUIRED_FOR_GAG_SYNTHESIS
DAZARD_UV_RESPONSE_CLUSTER_G2
NIKOLSKY_BREAST_CANCER_7P22_AMPLICON
WORSCHECH_TUMOR_REJECTION_UP
AMUNDSON_DNA_DAMAGE_RESPONSE_TP53
CHIANG_LIVER_CANCER_SUBCLASS_INTERFERON_UP
DAZARD_UV_RESPONSE_CLUSTER_G28
BOQUEST_STEM_CELL_CULTURED_VS_FRESH_DN
DASU_IL6_SIGNALING_UP
GENTILE_UV_LOW_DOSE_UP
NIKOLSKY_BREAST_CANCER_16P13_AMPLICON
FDR ≤ 0.05
FDR ≤ 0.25
-4
-2
0
2
Normalized Enrichment Score (NES)

Figure S2
